# Thiamine, gastrointestinal beriberi and acetylcholine signaling

**DOI:** 10.3389/fnut.2025.1541054

**Published:** 2025-04-09

**Authors:** Elliot Overton, Alina Emelyanova, Victoria I. Bunik

**Affiliations:** ^1^Objective Nutrients, Wareham, United Kingdom; ^2^Faculty of Bioengineering and Bioinformatics, Lomonosov Moscow State University, Moscow, Russia; ^3^Belozersky Institute of Physico-Chemical Biology, Lomonosov Moscow State University, Moscow, Russia; ^4^Department of Biochemistry, Sechenov University, Moscow, Russia

**Keywords:** thiamine, gastrointestinal beriberi, acetylcholine, intestinal ThDP-dependent enzymes, intestinal transport of thiamine, intestinal metabolism of thiamine, functional gastrointestinal disorder

## Abstract

Research has highlighted numerous detrimental consequences of thiamine deficiency on digestive function. These range from impaired gastric and intestinal motility to aberrant changes in pancreatic exocrine function, gastric acidity and disturbances in gut barrier integrity and inflammation. Thiamine and its pharmacological forms, as a primary or adjunctive therapy, have been shown to improve symptoms such as nausea, constipation, dysphagia and intestinal dysmotility, in both humans and animals. This review aims to explore molecular mechanisms underlying the therapeutic action of thiamine in gastrointestinal dysfunction. Our analysis demonstrates that thiamine insufficiency restricted to the gastrointestinal system, i.e., lacking well-known symptoms of dry and wet beriberi, may arise through (i) a disbalance between the nutrient influx and efflux in the gastrointestinal system due to increased demands of thiamine by the organism; (ii) direct exposure of the gastrointestinal system to oral drugs and gut microbiome, targeting thiamine-dependent metabolism in the gastrointestinal system in the first line; (iii) the involvement of thiamine in acetylcholine (ACh) signaling and cholinergic activity in the enteric nervous system and non-neuronal cells of the gut and pancreas, employing both the coenzyme and non-coenzyme actions of thiamine. The coenzyme action relies on the requirement of the thiamine coenzyme form – thiamine diphosphate – for the production of energy and acetylcholine (ACh). The non-coenzyme action involves participation of thiamine and/or derivatives, including thiamine triphosphate, in the regulation of ACh synaptic function, consistent with the early data on thiamine as a co-mediator of ACh in neuromuscular synapses, and in allosteric action on metabolic enzymes. By examining the available evidence with a focus on the gastrointestinal system, we deepen the understanding of thiamine’s contribution to overall gastrointestinal health, highlighting important implications of thiamine-dependent mechanisms in functional gastrointestinal disorders.

## 1 Introduction

Gastrointestinal diseases are highly prevalent worldwide and account for a significant portion of global disease burden (1) and healthcare costs (2). Globally, a substantial proportion of these diseases are linked to infectious and transmissible causes, whereas in developed countries the vast majority are non-communicable and are rising in prevalence (3). The most common diagnoses in gastroenterology are a group of disorders known as Functional Gastrointestinal Disorders (FGIDs). With growing recognition in recent years of the nervous system’s involvement in such disorders, they are now broadly defined as disorders of gut-brain interaction (4). According to the Rome IV criteria, such disorders can involve any combination of motility disturbance, altered mucosal and immune function, dysbiosis of gut microbiota, visceral hypersensitivity, and altered central nervous system processing (5). The estimated prevalence is 10–40% (6), although the actual prevalence is unknown (7). A recent global study shows that 49% of females and 37% of males meet the diagnostic criteria for at least one FGID (7). The five most prevalent FGIDs are irritable bowel syndrome, functional dyspepsia, functional constipation, functional diarrhea, and functional bloating/abdominal distention. However, the FGID classification also includes a diverse range of other disorders, many of which exhibit overlapping clinical features. These include epigastric pain syndrome, functional dysphagia, heartburn, reflux hypersensitivity, belching disorder, bloating/distention, centrally mediated abdominal pain, fecal incontinence, and others.

The molecular and cellular mechanisms underpinning the pathophysiology of FGIDs are complex and have been reviewed elsewhere (5). Prominent features include epithelial barrier dysfunction, delayed or accelerated gastrointestinal transit due to abnormal function of smooth muscles, dysfunctional enteric nervous system and/or immunity, gut microbial dysbiosis, bile acid malabsorption, and alterations in the gut-brain axis (8–12). Therapeutic interventions include pharmacologic agents, dietary and lifestyle changes, probiotics, antibiotics, fecal microbial transplant, and stress management. Pharmacological treatments range from prokinetics and antispasmodics to centrally acting neuromodulators, albeit with varied success (13). As research continues to unearth a multitude of pathophysiological mechanisms involved in FGIDs, it is important to reveal specific primary causes. Similar symptoms may originate from different impairments, but therapies mitigating the primary impairment, rather than the convergent symptoms, are the most efficient ones (14).

Publications on FGIDs, which are ameliorated by administration of thiamine (vitamin B1) (15–23), suggest that thiamine insufficiency in the gastrointestinal system and/or its innervation may contribute causally to the development of these disorders, also in the absence of systemic thiamine deficiency. Our review aims at understanding the molecular mechanisms underlying this potential of thiamine to counteract many pathological alterations commonly observed in FGIDs. In addition to thiamine’s role as a precursor of an essential coenzyme in energy production from glucose oxidation (24), contribution to gastrointestinal function of the non-coenzyme action of thiamine (25, 26), presumably regulating not only the coenzyme-dependent pathway of acetylcholine (ACh) biosynthesis (27), but also ACh signaling at neuromuscular junctions (28) and in non-neuronal cells (29), is considered. Our focus on an intestine-expressed set of proteins which are well-known or suggested to be involved in transport, transformations and action of thiamine and its derivatives, underscores the significance of polar distribution of different thiamine transporters to organize the nutrient flux in enterocytes. We draw attention to various factors that may perturb enterocytic balance between the thiamine influx from the lumen and systemic delivery to other tissues, providing insights into the molecular mechanisms of local thiamine insufficiency in gastrointestinal system. Although this form of thiamine deficiency, which we call gastrointestinal beriberi, is initially limited to the gastrointestinal system, it mostly progresses to systemic thiamine deficiency, often triggered by stresses of different etiologies. That is why the distinction between specific gastrointestinal beriberi and the gastrointestinal manifestations of systemic beriberi resulting from gastrointestinal dysfunction remain unclear and are not well defined. However, in line with the existing classification of beriberi affecting nerves (dry beriberi) and cardiovascular system (wet beriberi), it is plausible to use the term gastrointestinal beriberi in an analogous, tissue-directed, way.

Our analysis of the gastrointestinal beriberi mechanisms employs, in particular, case studies of patients after bariatric surgery – a well-defined group, often developing systemic thiamine deficiency. Using available data on the time-dependent transcriptomic changes in enterocytes of these patients, we demonstrate long-term perturbations in their thiamine metabolism, which may be of diagnostic significance. Existence of genetic variants of thiamine-dependent proteins, insufficient molecular identification of these proteins, as well as the age- and sex-dependent differences in thiamine metabolism (30), may contribute to the observed variety of clinical manifestations of thiamine deficiency (31, 32). Clearly, this may also affect the outcomes of systemic and meta-analyses of thiamine’s effects in different populations and patient groups (32). As a result, our review calls upon more attention to personalized approaches in characterization of individual thiamine homeostasis, based on the knowledge of the thiamine-dependent proteins and their tissue-specific features and functions.

## 2 Thiamine (vitamin B1) and its metabolic significance

**Figure 1:**
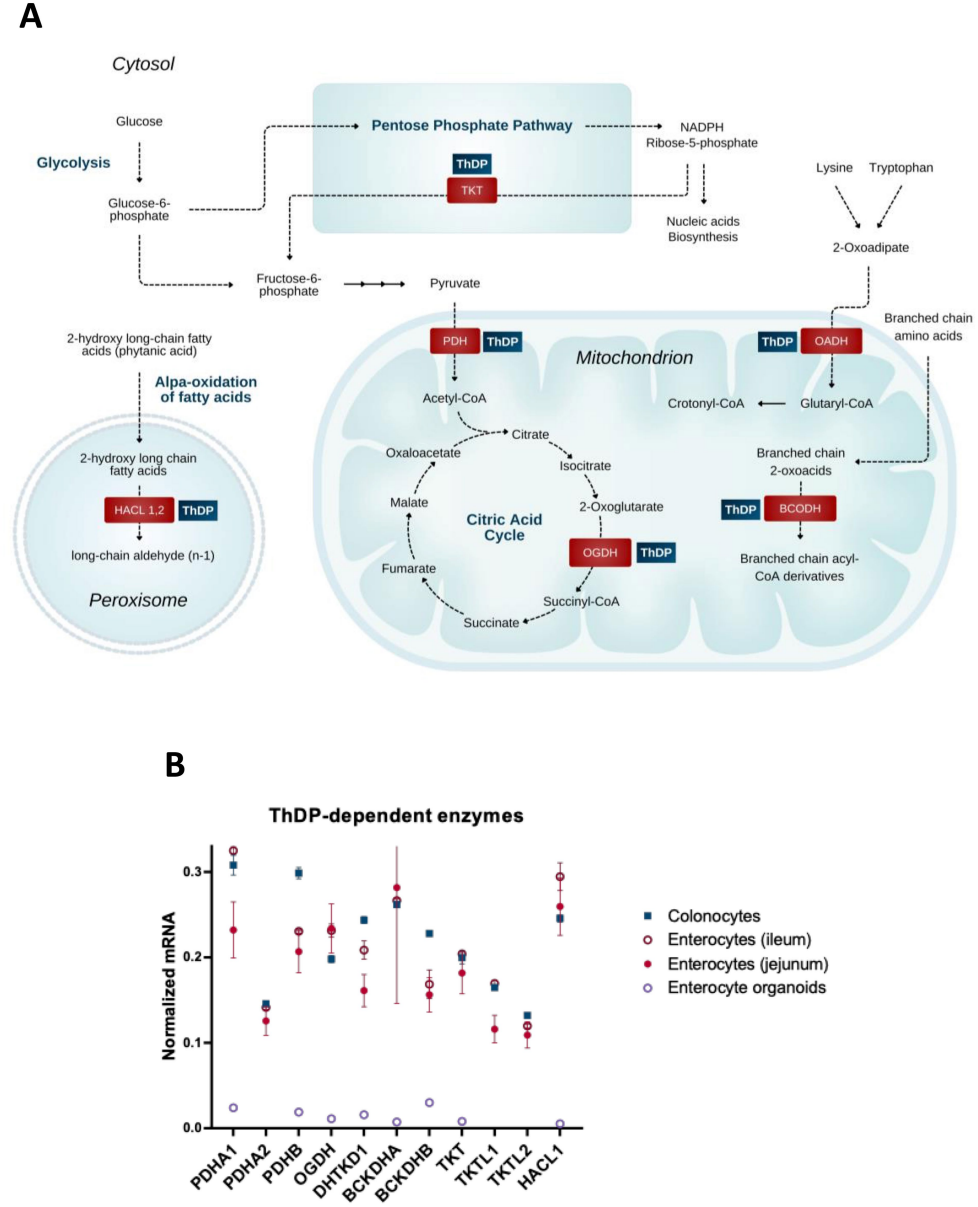
Coenzyme role of ThDP in metabolism of the gut cells. **(A)** Metabolic pathways involving enzymes that use the coenzyme derivative of thiamine, thiamine diphosphate (ThDP). **(B)** The relative abundance of mRNAs for the ThDP-dependent enzymes in different cells of intestine (colonocytes, enterocytes of ileum, enterocytes of jejunum) and in enterocyte organoids. The transcript signals for the genes of interest are normalized to the sum of the average mRNA signals of GAPDH, ACTB and TUBA1A as described earlier (149). The signals of these mRNAs for the three transcripts used for the normalization, are comparable, producing similar normalization ratios of the transcripts of interest across the different GEO datasets used. Transcriptomics data is taken from the GEO database. Identificators of the assessed experiments are: colonocytes experiments GSE13367 [Platform GPL570, (HG-U133_Plus_2) Affymetrix Human Genome U133 Plus 2.0 Array, 10 datasets: GSM337520, GSM337526, GSM337529, GSM337530, GSM337532, GSM337533, GSM337537, GSM337539, GSM337540, GSM337544] and GSE30292 [Platform GPL570, (HG-U133_Plus_2) Affymetrix Human Genome U133 Plus 2.0 Array, 3 datasets: GSM750882, GSM750883, GSM750884]; jejunum enterocytes experiments GSE214758 [Platform GPL20795, HiSeq X Ten (*Homo sapiens*), 9 datasets: GSM6615629, GSM6615631, GSM6615633, GSM6615637, GSM6615641, GSM6615643, GSM6615645, GSM6615647, GSM6615649], GSE113819 {Platform GPL17586 (HTA-2_0) Affymetrix Human Transcriptome Array 2.0 [transcript (gene) version], 5 datasets: GSM3120595, GSM3120597, GSM3120599, GSM3120601, GSM3120603} and GSE30292 [Platform GPL570, (HG-U133_Plus_2) Affymetrix Human Genome U133 Plus 2.0 Array, 2 datasets: GSM750891, GSM750892]; ileum enterocytes experiment GSE30292 [Platform GPL570, (HG-U133_Plus_2) Affymetrix Human Genome U133 Plus 2.0 Array, 3 datasets: SM750888, GSM750889, GSM750890]; enterocyte organoid experiment GSE242765 [Platform GPL18573, Illumina NextSeq 500 (*Homo sapiens*), 1 dataset GSM7770156]. Normalized mRNA levels from all the datasets for the same cell type are averaged, and the data are shown as mean ± SEM.

Thiamine is an essential water-soluble vitamin, naturally occurring in a wide variety of foods. Rich dietary sources include different types of meat, legumes and whole grains. In the human body, the major intracellular derivative of thiamine is its diphosphorylated form, thiamine diphosphate (ThDP). This essential coenzyme of ThDP-dependent enzymes is absolutely necessary not only for oxidative glucose metabolism, but also for coupling metabolic pathways of carbohydrates and amino acids, as well as for controlling *de novo* synthesis of major neurotransmitters, ACh and glutamate, from glucose. The control is determined by ThDP-dependent biosynthesis and/or distribution of the neurotransmitter precursors acetyl-CoA and 2-oxoglutarate, formed upon the oxidation of the glycolytic product pyruvate in the TCA cycle (Figure 1).

The mammalian ThDP-dependent enzymes (24) include cytosolic transketolase (TKT) participating in the pentose phosphate pathway; the mitochondrial multienzyme complexes of 2-oxo acids, i.e., of pyruvate dehydrogenase (PDH), 2-oxoglutarate dehydrogenase (OGDH), 2-oxoadipate dehydrogenase (OADH) and branched chain 2-oxo acid dehydrogenase (BCODH), linked to amino acids through their transamination to the 2-oxo acids; and peroxisomal 2-hydroxy acyl-CoA lyase (HACL) participating in the *α*-oxidation of long and very long chain 3-methyl or 2-hydroxy even-chain fatty acids (33).

TKT, as part of the cytosolic pentose phosphate pathway, along with the mitochondrial multienzyme complexes of pyruvate (PDHC) and 2-oxoglutarate dehydrogenases (OGDHC), are essential for energy production from glucose oxidation. The oxidation-generated reducing equivalents accumulate as cytosolic NADPH produced by the pentose phosphate pathway, and mitochondrial NADH, produced by 2-oxo acid dehydrogenases. In addition, the function of TKT in the pentose phosphate pathway supports the generation of phosphoribose for nucleic acid synthesis. PDHC links cytosolic glycolysis to the mitochondrial TCA cycle by oxidizing pyruvate, the end product of glycolysis. The PDHC-catalyzed reaction generates not only NADH, which is oxidized in the mitochondrial respiratory chain, but also acetyl-CoA, which feeds into the TCA cycle and is a precursor of acetylcholine (ACh) in cholinergic cells. OGDHC catalyzes the rate-limiting step of the TCA cycle. The substrate of the OGDHC reaction, 2-oxoglutarate, is a precursor for glutamate biosynthesis from glucose. The OGDHC reaction product succinyl-CoA provides for the only mitochondrial phosphorylation reaction of ADP to ATP, at the substrate-level, i.e., phosphorylation beyond mitochondrial respiratory chain.

In addition to the ubiquitous ThDP-dependent enzymes, enterocytes express a number of the ThDP-dependent isoenzymes, i.e., the enzymes with similar catalytic functions, encoded by separate genes. These include transketolase-like 1 and 2 proteins, TKTL1 and TKTL2, as well as the ubiquitous (PDHA1) and testis-specific (PDHA2) isoenzymes of the *α*-subunit of PDH (PDHA) (Figure 1B). Deciphering physiological roles of isoenzymes is often challenging. Inactivation of TKTL1 aggravates colitis in a murine knockout model (34, 35). Isoenzymes of HACL1 and HACL2 are shown to provide *α*-oxidation of 3-methyl and 2-hydroxy long chain fatty acids, respectively, with HACL2 playing an important role in ceramide formation in the stomach (33). Isoenzymes of OGDH are the brain-specific OGDH-like protein (OGDHL) and OADH, encoded by OGDHL and DHTKD1 genes, correspondingly (Figure 1). OGDH and OGDHL differ in their regulatory properties, including 2-oxoglutarate saturation (36). OGDH(L) and 2-oxoadipate dehydrogenase (OADH) have different substrate specificity, preferring 2-oxoglutarate and 2-oxoadipate, correspondingly (37, 38). The OGDH isoenzymes encoded by OGDHL and DHTKD1 genes, link eosinophilic esophagitis to mitochondrial dysfunction (39).

Functional significance of the ThDP-dependent enzyme isoforms, which are the enzyme variants arising from alternative splicing of transcripts from a single gene, or through post-translational modifications, is studied even less than that of isoenzymes. An exception is the established regulatory significance of the OGDH splice variants lacking Ca^2+^-dependent regulation. Unlike skeletal muscle and heart, which predominantly express Ca^2+^-sensitive isoforms, other tissues, in particular pancreatic islets, have significant expression of Ca^2+^-independent OGDH isoforms, which are thought to be involved in the distribution of 2-oxoglutarate flux to oxidation in the TCA cycle and glutamate biosynthesis (40).

As mentioned above, the coenzyme role of thiamine is important for metabolism of *α*-amino acids, as they are transaminated to 2-oxo acids. In addition to PDHC and OGDH(L)C, this action of thiamine is mediated by two other ThDP-dependent multienzyme complexes, i.e., those of branched chain 2-oxo acid dehydrogenase (BCODH) and OADH, functioning in the pathways of degradation of branched-chain *α*-amino acids, and lysine and tryptophan, respectively (Figure 1). The corresponding acyl-CoAs generated by these complexes undergo additional transformations before entering the TCA cycle in the form of acetyl- or succinyl-CoA. As a result, catalytic function of the multienzyme complexes of 2-oxo acid dehydrogenases may provide energy from oxidation of both carbohydrates and amino acids. Thiamine-dependent metabolic regulation is important for optimizing the energy source: by increasing efficiency of glucose oxidation, thiamine prevents excessive degradation of amino acids (41).

In recent years, the role of different acyl-CoAs in the posttranslational acylation of protein lysine residues has acquired increasing attention (42). Histone acylations are considered to be especially important for intestinal epithelium adaptations to environmental signals (43). Remarkably, nuclear localization of the 2-oxo acid dehydrogenase complexes has recently been discovered, in addition to their traditional mitochondrial localization (44–47). This finding is in good accordance with independent research that acylations of both metabolic proteins and histones depend on the function of 2-oxo acid dehydrogenase complexes (48, 49). The changes in histone acylation, mediated by ThDP-dependent 2-oxo acid dehydrogenase complexes, provide a mechanism for thiamine-dependent transcriptional regulation.

Positioning of ThDP-dependent 2-oxo acid dehydrogenase complexes at the intersections of major pathways of central metabolism and transcriptional regulation endows these systems with a functional role of “signaling hubs,” regulating multiple cellular processes, such as intracellular redox status, growth, protein signaling, and calcium homeostasis (50–52).

Expression of the considered ThDP-dependent enzymes and isoenzymes in enterocytes (Figure 1B) underscores both the common and isoenzyme-specific dependence of gastrointestinal metabolism on thiamine.

## 3 Gastrointestinal beriberi

As animals do not synthesize thiamine, their thiamine need is satisfied by dietary intake and biosynthesis by gut microbiota. Thus, gastrointestinal dysfunction impairing the absorption of nutrients inevitably results in insufficient organismal thiamine levels, i.e., thiamine deficiency (TD). Indeed, the most well-known TD state is Wernicke-Korsakoff syndrome, first described by Wernicke in a female whose digestive tract was severely damaged by sulfuric acid (53). On the other hand, gastrointestinal disturbances are critical early indicators of severe thiamine deficiency causing pediatric Wernicke Encephalopathy (54).

An estimated 2–3% of the body’s thiamine may originate from microbial synthesis within the colon, although its precise nutritional significance remains to be fully understood. Studies on the gut microbiome have identified distinct genera of bacteria capable of synthesizing thiamine, including *Lactobacillus*, *Bifidobacterium*, *Escherichia coli*, *Enterococcus*, and *Clostridium* (55, 56). Furthermore, high expression of genes involved in thiamine biosynthesis and transport are prevalent in *Prevotella*, *Desulfovibrio* (57), and *Bacteroides* (58). On the other hand, the microbiome houses many genera of bacteria that fail to grow in the absence of thiamine (59), and depend on its external sources (60). The abundance of one such family, *Ruminococcaceae*, is positively correlated with thiamine intake in humans. Dietary thiamine restriction in rodents also decreases relative abundance of this family, which is accompanied by reduced fecal butyrate concentrations (61). ThDP is used as a cofactor for the microbial enzyme pyruvate-ferredoxin oxidoreductase, which catalyzes the conversion of pyruvate to acetyl-CoA in the pathway for butyrate synthesis (56). Hence, bacteria require thiamine for production of short-chain fatty acids, which play important anti-inflammatory and signaling roles in the gut. It is therefore not surprising that thiamine from dietary sources may alter the composition of microbes in the gut, and a deficit of thiamine may result in bacterial dysbiosis (58, 59).

Remarkably, in a trial using high-dose thiamine for fatigue related to inflammatory bowel disease (IBD), the relative abundances of *Faecalibacterium prausnitzii*, a member of the *Ruminococcacea* family, and *Roseburia hominis*, a member of the *Lachnospiraceae* family, inversely correlate with fatigue severity both pre- and post-treatment with thiamine (62).

When TD becomes severe and chronic, it is known as beriberi. Dry beriberi affects the central nervous system (CNS) and peripheral nerves, whereas wet beriberi involves the cardiovascular system. In 2004, Dr. Michael Donnino introduced the term “gastrointestinal beriberi” (63) to describe a distinct clinical entity caused by TD in the gastrointestinal system. It is broadly defined as a combination of several possible symptoms: anorexia (lack of appetite), nausea, unexplained vomiting, abdominal distention, constipation, reflux and epigastric pain, and occasionally intestinal paralysis. Since its original definition in the medical literature, multiple case reports have been published by independent researchers (Table 1). Although manifestations of neurological dysfunction often point to TD and may sometimes accompany gastrointestinal beriberi (17, 64), it has been also noted that chronic mild deficiency may present with gastrointestinal symptoms under entirely normal neurological exams in well-nourished, non-alcoholic patients (65–68). In one of the early reports cited by Donnino (69), severe manifestations of gastrointestinal beriberi are also preceded in time by much milder symptoms. These include abdominal distension, belching, and alternating constipation and diarrhea. Furthermore, early into the study, all participants exhibited achlorhydria or hypochlorhydria, delayed gastric emptying, and reduced intestinal motility. Similarly, early gastrointestinal symptoms - such as a full sensation in the epigastrium, gastric reflux, hypochlorhydria, impaired gastric and intestinal motility, and constipation - are reported by Shimanoza and Katsura in 30–50% of patients with TD (70). In another study, thiamine therapy leads to the disappearance of both gastrointestinal (dysphagia) and CNS/cardiovascular (dyspnea and blurred vision) symptoms. However, after thiamine is discontinued, dysphagia re-appears at the time when other symptoms are not observed (71). One case report describes the onset of dysphagia and gastroparesis in a patient with Crohn’s disease 2 months before developing Wernicke encephalopathy, which resolved with high dose thiamine (72). A recent analysis (19) has found gastrointestinal symptoms in 46 out of 52 patients with diagnosed TD. In some cases, symptoms precede the classical neurological signs by up to several months, suggesting that digestive dysfunction may, in fact, be an early indicator of TD before progressing to other bodily systems. Dysphagia, or esophageal dysmotility, is common after bariatric surgery, known to be associated with TD, and is restored following thiamine administration (73, 74). A high prevalence of constipation and gastrointestinal paralysis is also associated with TD (70). Numerous reports demonstrate significant impairments in intestinal motility (small and large) in TD subjects, which normalizes with thiamine repletion (18, 75–78). Dietary thiamine intake is inversely associated with constipation (79). Abnormal gastric acidity is reported to be one of the most common and early signs of TD (70). In this study, four patterns of acid secretion are identified in response to thiamine administration: (i) Hypoacidity which gradually improves with thiamine repletion; (ii) Hypoacidity which shifts to high acidity in the recovery phase after thiamine administration, followed by eventual normalization; (iii) Hyperacidity in the early stage of TD, followed by normalization with thiamine therapy; (iv) Hyperacidity, followed by low acidity during the recovery phase after thiamine administration, which normalizes later. More recently, aberrant changes in gastric output have been demonstrated in TD. Achlorhydria is found in both human (80–83) and animal (84) studies. TD is also known to induce gastric ulceration (85). Epigastric pain, early satiety, and gastroesophageal reflux are common initial symptoms of TD and gastrointestinal beriberi (19, 70). Based on modern diagnostic criteria, this combination of symptoms could now be broadly classified as functional dyspepsia, which is often associated with gastric hypoacidity (86). Thiamine in conjunction with other therapies has been used to successfully treat functional dyspepsia (87).

**Table 1:** Therapeutic action of thiamine administration in human and animal studies of FGIDs.

GI symptoms resolved after thiamine administration	Intervention	Sample size
Humans		
Nausea, vomiting, abdominal pain, anorexia in patients with Wernicke encephalopathy (19)	I.v. infusions of 300-600 mg of thiamine for 5–10 days, followed by oral thiamine at 100-300 mg	*n* = 42
Nausea, vomiting, anorexia preceding Wernicke encephalopathy (17)	Single i.v. injection of 1,000 mg of thiamine	Case report (*n* = 1)
Constipation in patients with thiamine deficiency after Roux-en-Y Gastric Bypass. Decreased blood levels of thiamine with increased folate levels are shown (18)	I.m. injections of 100–200 mg of thiamine monthly, some patients taking 200 mg oral thiamine daily	*n* = 11
Perturbed intestinal motility/gas release in post-hysterectomy patients (16)	I.m. injection of 100 mg thiamine for 2 days	*n* = 80
Dysphagia and gastroparesis preceding Wernicke encephalopathy in Crohn’s disease (72)	I.v. injections of 200 mg thiamine three times, followed by I.m. injections of 100 mg/day for several months	Case report (*n* = 1)
Intestinal paralysis in post-hysterectomy patients (20)	I.m. injection of 100 mg thiamine for 3 days	*n* = 60
Animals		
Ruminal epithelial barrier dysfunction, oxidative stress and apoptosis, induced by high-concentrate diet in goats (22)	Thiamine 200 mg/kg dry feed for 12 weeks	*n* = 8
Perturbed colonic integrity and mucosal inflammation, induced by high-concentrate diet in goats (21)	Thiamine 200 mg/kg dry feed for 12 weeks	*n* = 8
Experimental constipation induced by atropine and papaverine in rats (23)	S.c. injection of 100 mg/kg TTFD	*n* = 4
Ulcerative colitis rat model (15)	I.p. injection of 20 mg/kg thiamine per day for 5 days	*n* = 6

Thus, a wide range of gastrointestinal disorders respond positively to thiamine administration. The therapeutic action of thiamine administration in human and animal studies are summarized in Table 1.

An important factor for digestion and overall gastrointestinal health is pancreatic function (88). Of note, the pancreas has a high thiamine content (89) which it maintains through constant uptake from circulation (90). TD is associated with a dramatic reduction of pancreatic thiamine content by up to 75% (89). Depletion of pancreatic thiamine can result in oxidative stress (91), which is considered to be a driving factor in development of pancreatitis. Furthermore, some researchers speculate that thiamine depletion in the pancreas may be a necessary antecedent for pancreatic inflammation (91). Indeed, chronic alcoholism and nicotine consumption are independent risk factors for pancreatitis, and both inhibit pancreatic thiamine uptake via downregulation of thiamine transporters (90). A Spanish review published in 1944 reports that hyposecretion of pancreatic enzymes is a common feature of TD (92), and administration of thiamine in children could increase the release of the pancreatic enzymes trypsin, amylase and lipase (93). Animal research also highlights substantial abnormalities in pancreatic function during TD, evidenced by a reduction in stored pancreatic protein and digestive enzyme content, contrasted by an abnormally large enzyme secretion (94). Acute pancreatitis and encephalopathy are recently reported as a consequence of TD and successfully treated with thiamine administration (95).

As a result, thiamine is known to positively influence perturbed gastrointestinal function. In many cases, this positive influence occurs when CNS symptoms of TD are absent, pointing to the gastrointestinal system as the primary site of TD. The findings suggest that TD restricted to the gastrointestinal tract may occur, while other TD-sensitive systems, such as the neurological and cardiovascular systems, do not display their specific symptoms. That said, chronic impairment of thiamine availability in the gastrointestinal system should also reduce resilience of other body systems. Obvious neurological and/or cardiovascular symptoms may represent a culmination of gastrointestinal beriberi, probably triggered by challenges that increase metabolic demands or decrease intracellular transport of thiamin, such as stress, infection, trauma, drug administration. For instance, a case study describes persistent gastrointestinal beriberi followed by the onset of neurological symptoms in a male patient after one session of heavy drinking (17). Metformin, a widely used antidiabetics entering cells through the thiamine transporters, is known to reduce intestinal thiamine content in mice (96). Thus, different comorbidities may transition a vulnerable state of gastrointestinal beriberi—where TD is confined to the gastrointestinal tract, but is not obviously affecting other body systems—into dry or wet beriberi, characterized by specific symptomatic manifestations where potentially life-threatening situations promote the diagnosis of TD.

In conclusion, TD limited to the gastrointestinal system may be an overlooked and underdiagnosed cause of the increasingly common gastrointestinal disorders encountered in modern medical settings. Left unattended, it may progress to wet or dry beriberi, most often observed as Wernicke encephalopathy. However, how is it possible that the gastrointestinal system—which, unlike other systems, is directly exposed to nutrients—suffers from this nutrient deficiency more than the rest of the body? The following sections examine the available evidence on molecular mechanisms of thiamine transport, metabolism, and function in the gastrointestinal system, offering insights for the interpretation of medical studies on thiamine-responsive gastrointestinal disorders that highlight mechanistic connections between common disorders of gastrointestinal tract and TD.

## 4 Thiamine metabolism in intestinal cells

### 4.1 Thiamine transport in brush border and basolateral membranes of enterocytes

**Figure 2:**
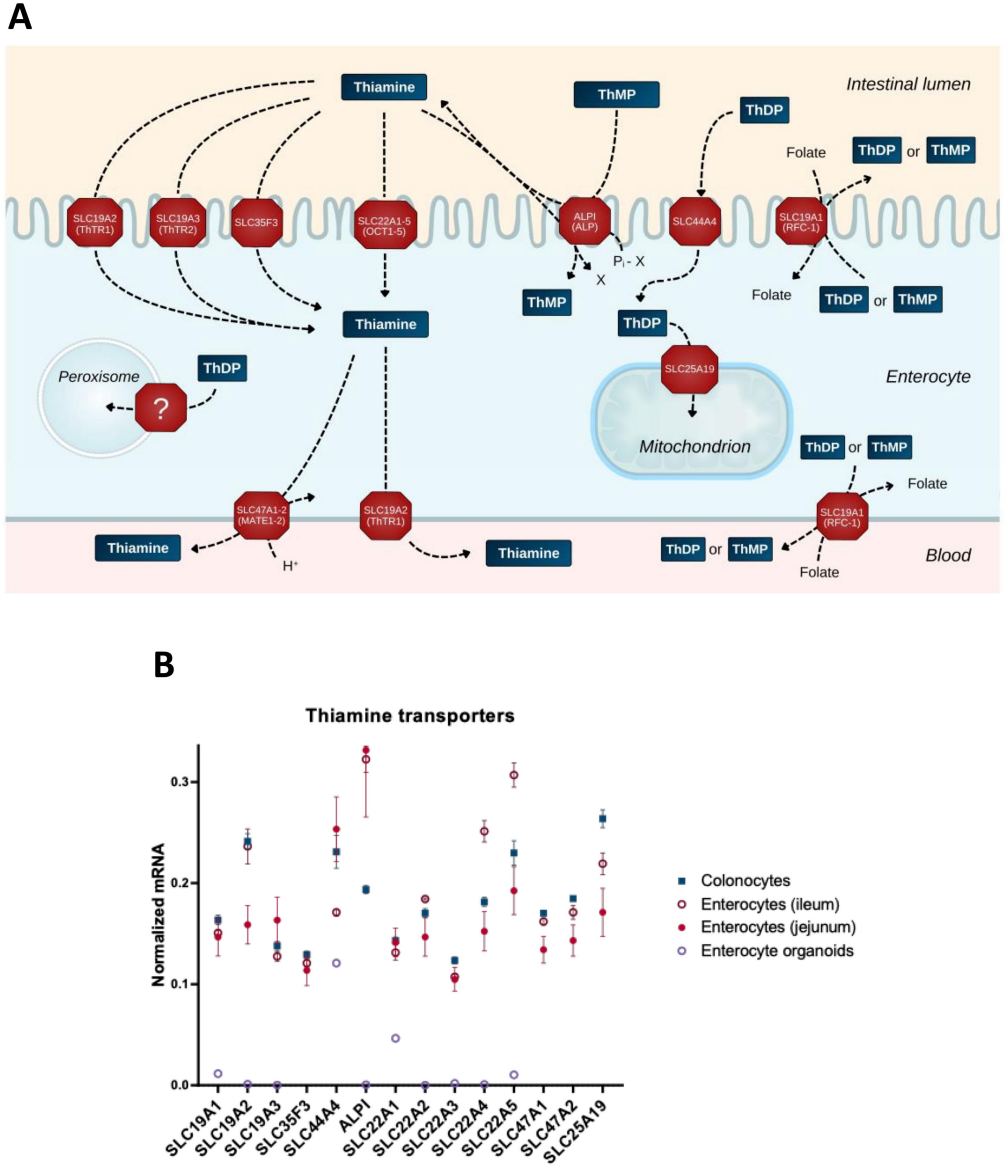
Transporters of thiamine and its derivatives in the gut cells. **(A)** Schematic presentation of the transport processes through transporters expressed in the gut cells. **(B)** The relative abundance of mRNAs for the transporters of thiamine and its derivatives in different intestinal cells (colonocytes, enterocytes of ileum, enterocytes of jejunum) and in enterocyte organoids. The normalized mRNA signals are calculated as described in the legend to Figure 1 using the same datasets. The data are shown as mean ± SEM.

Due to its hydrophilic nature, thiamine is not membrane-permeable, with its entry into the cell depending on membrane proteins dedicated to thiamine transport. Enterocytic absorption of dietary thiamine from the lumen and further transport of thiamine from the intestine to the blood occur via active transport through a number of transporters (Figure 2A). Thiamine transport capacity in human intestinal biopsy samples is highest in the duodenum, followed by the colon and stomach (97). Although THTR1 and THTR2 transporters, encoded by the SLC19A2 and SLC19A3 genes, respectively, are the most widely known and well-characterized, a number of newly characterized transporters of thiamine and its phosphates have been added to the list in recent decades. Figure 2 shows those expressed in intestine (A) along with their relative expression in enterocytes, their organoids and colonocytes (B).

Thiamine transporter SLC35F3 has been identified in studies investigating its variant, which is associated with human hypertension and lower levels of erythrocytic thiamine; expression of the protein in *Escherichia coli* increases thiamine transport (98). Thiamine is also transported through OCT1 (SLC22A1) (96) and OCT2 (SLC22A2) (99). Although the substrate specificity of other OCT family members is insufficiently characterized, their principal ability to transport organic cations justifies inclusion of SLC22A3–5 in Figure 2.

Intestinal alkaline phosphatase (ALPI, Figure 2) plays an essential role in the absorption of dietary B vitamins including thiamine (100). This enzyme not only exhibits phosphatase activity, dephosphorylating thiamine phosphates in the lumen, but may also transphosphorylate extracellular thiamine to intracellular thiamine monophosphate (ThMP), at the expense of intracellular phosphate donors such as beta-glycerophosphate or creatine phosphate (101). Transporters of MATE (multidrug and toxin extrusion) family (SLC47A) extrude thiamine in exchange for a proton; SLC47A1 and SLC47A2-K are characterized by a Km for thiamine in micromolar range (102), supporting the physiological relevance of thiamine extrusion by these transporters (Figure 2A).

Differences in the net intestinal transport of thiamine have been studied by Rindi and co-workers using basolateral and brush border membrane vesicles (100, 103, 104). However, apart from the SLC19A transporter family, little is known about the polar distribution of transporters in enterocytes and colonocytes. The gut cells have an apical, or brush border, membrane facing the lumen, and a basolateral membrane facing the circulatory system. This polarity is in accord with the dual function of intestinal epithelium: the same cells absorb nutrients from the lumen through the brush border membrane and deliver them to the blood through the basolateral membrane (Figure 2A). Available data on the polarity are taken into account in Figure 2A. These refer to the excretory function of MATE transporters (102, 105, 106) and the identification of SLC19A2 on both membrane types in epithelial cells (107). In contrast, the SLC19A3 protein is shown to localize specifically to the apical (brush border) membrane (108). This transporter is well-characterized in terms of its structure and function, with a number of drugs decreasing thiamine transport through SLC19A3 (109). With the brush border membrane directly exposed to drugs inhibiting thiamine influx through SLC19A3, and nutrient efflux through the basolateral membrane to satisfy permanent systemic demands for thiamine, the steady-state concentration of thiamine in the gut epithelium may be decreased. The disbalance between the inhibited enterocytic influx and unchanged or increased systemic demand may cause specific vulnerability of the gut epithelium to toxic effects of the thiamine-competitive drugs targeting SLC19A3. As a result, gastrointestinal TD may develop and exist as a steady-state, providing other tissues with thiamine levels sufficient for normal conditions, but inadequate for metabolic challenges.

As luminal ThDP from the diet is supposed to be hydrolyzed by ALPI, while ThDP is known to be produced inside the cell, the role of SLC44A4 in transporting extracellular ThDP has been enigmatic. Single nucleotide polymorphisms in SLC44A4 are key risk factors for ulcerative colitis in a collection of different studies (110–113). In view of the high expression of this transporter in colon, it has been proposed to participate in absorption of the microbiota-generated ThDP (25). However, this view leaves unanswered the question why microbes would extrude ThDP to the lumen. Besides, the transporter is also expressed in other tissues, and independent data (114–116) show a less drastic difference between human colonocytes and enterocytes in its expression (Figure 2B), compared to the initial finding of a 10-fold higher expression in the colon relative to other regions of gastrointestinal tract in humans (117). On the other hand, according to recent structure–function characterization of reduced folate transport through SLC19A1, ThDP is the best substrate to be exchanged for folate (118). The participation of ThDP in cellular folate absorption (Figure 2A) endows SLC44A4 with a physiologically relevant function to return ThDP that is extruded in exchange for folate, back to the cell (Figure 2A). Employment of the ThDP-folate exchange by microbes may explain the high SLC44A4 expression in the colon. Yet, the expression of this transporter in different parts of the gut (Figure 2B) does not exclude its possible participation in the gut’s absorption of ThDP from the diet, especially given the presence of other folate transporters on the brush border membrane of enterocytes, i.e., those encoded by SLC46A1, FOLR1, and FOLR2 (119).

Interestingly, males display lower plasma folate levels than females (120–122), and only males demonstrate a positive correlation between the folate deficiency risk scores calculated from the polymorphisms in the folate pathway genes SLC19A1 and MTHFR (123). At the same time, it is known that thiamine intake is higher in males vs. females (124). The opposite sex-dependent differences in the levels of folate and thiamine highlight the physiological significance of the folate/ThDP exchange through SLC19A1. This is further supported by a study of the SLC19A1 variant rs1051266:G (125). In model experiments employing HEK293 cells in a thiamine-deficient medium, the SLC19A1 variant rs1051266:G exhibits a strong decrease in ThDP efflux compared to the canonic SLC19A1 sequence rs1051266:A. Association of the SLC19A1 variant rs1051266:G with Wernicke-Korsakoff encephalopathy (125), a condition known to develop due to the thiamine deficiency in the brain, underscores the role of SLC19A1 in delivering not only folate but also thiamine to CNS (28). Both SLC19A1 and SLC19A2 deliver vitamins to systemic tissues (119), correspondent to their locations in the basolateral membrane (Figure 2). ThDP transport through the folate transporter encoded by SLC19A1 is further supported by cases of thiamine-responsive dysphagia that have been observed at normal laboratory values of blood thiamine, but increased serum folate levels (71). The blood transports thiamine or its monophosphate (ThMP), both of which also penetrate the blood–brain barrier (26, 126) through saturable transporters (127).

### 4.2 Interconversions of thiamine and its natural derivatives in intestinal cells

**Figure 3:**
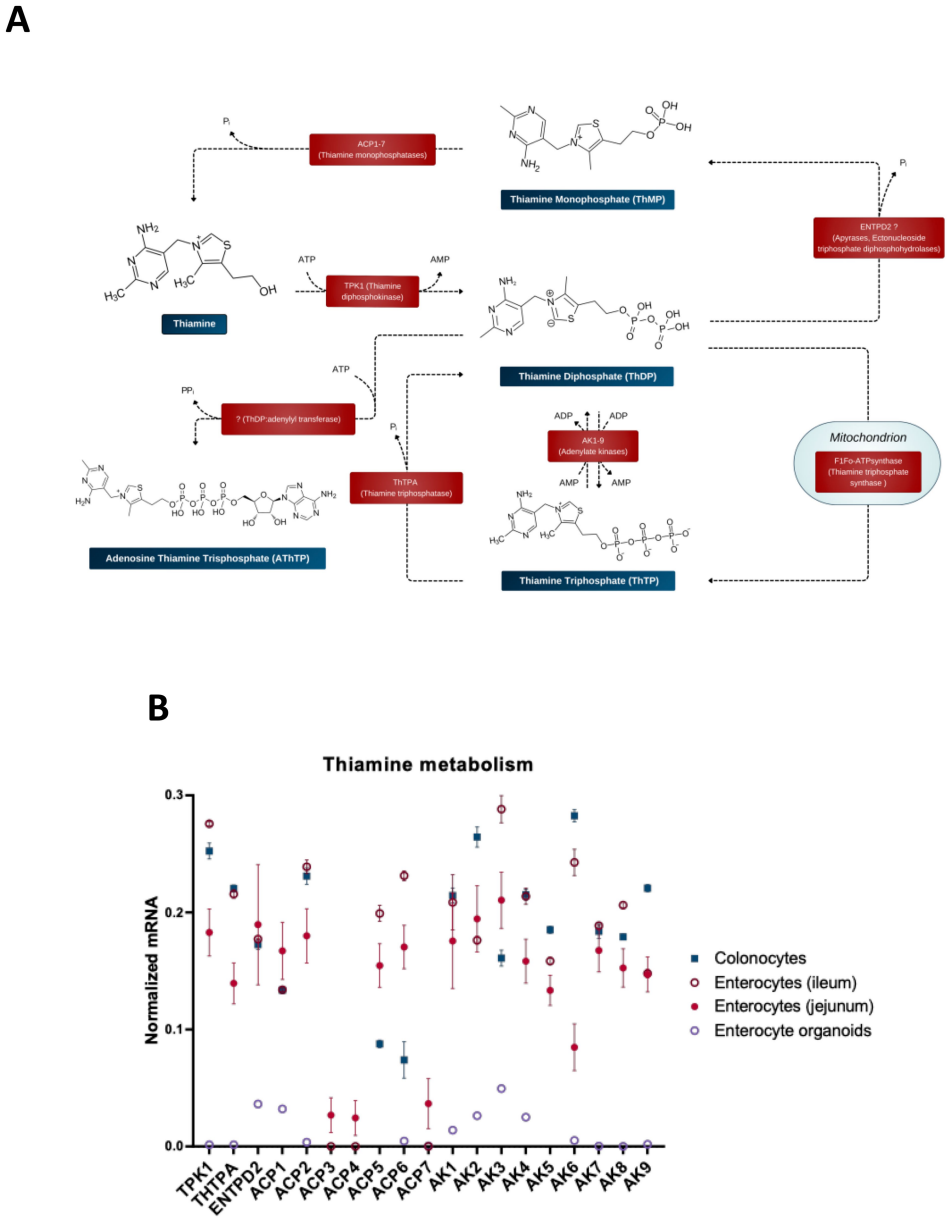
Enzymatic transformations of thiamine and its derivatives in the gut cells. **(A)** Schematic presentation of the reactions and available information on their catalysts. **(B)** The relative abundance of mRNAs for the enzymes of thiamine metabolism in different intestinal cells (colonocytes, enterocytes of ileum, enterocytes of jejunum) and in enterocyte organoids. The normalized mRNA signals are calculated as described in the legend to Figure 1 using the same datasets. The data are shown as mean ± SEM.

To perform its coenzyme function (Figure 1A), thiamine or ThMP entering the cells (Figure 2A) must be transformed into the coenzyme form, ThDP. Animals synthesize ThDP by diphosphorylation of thiamine in the thiamine diphosphokinase (or thiamine pyrophosphokinase, TPK)-catalyzed reaction (Figure 3A). Another well-characterized protein of thiamine metabolism is thiamine triphosphatase, encoded by ThTPA gene, which hydrolyzes thiamine triphosphate (ThTP) to ThDP (128, 129). The enzymes catalyzing other transformations of thiamine derivatives are not unambiguously identified. Given the poorly characterized specificity of enzymes involved in thiamine transformation and the often unknown roles of genomics-identified isoenzymes, all human isoenzymes are included in Figure 3 when there is evidence that at least one isoenzyme can catalyze the reactions with thiamine or its derivatives. In doing this, we would like to draw attention to possible roles of the isoenzymes, as shown in Figure 3, in thiamine metabolism. This may further help decipher their physiological significance, e.g., when the data on phenotypes of the mutated isoenzymes in humans may appear.

ThMP kinase, which produces ThDP through ThMP phosphorylation and is known in thiamine-synthesizing organisms, has not been identified in animals. Accordingly, to be transformed into ThDP by TPK, intracellular ThMP should first be dephosphorylated. ThMP hydrolysis is known to be catalyzed by prostatic acid phosphatase ACP3, which is localized to plasmatic membrane and linked to the antinociceptive action of thiamine and its derivatives (130–132). Cells of the gut express all isoenzymes of acid phosphatase (ACP), i.e., ACP 1–7 (Figure 3B). According to the UniProt database, these isoenzymes have diverse locations and substrate specificities, acting on protein phosphotyrosine residues and a number of alkyl, aryl and acyl orthophosphates of low molecular mass. ACP1 has cytosolic location, whereas ACP2 and ACP5 are lysosomal enzymes. ACP4 is a transmembrane protein with 50% homology to the prostatic and lysosomal acid phosphatases, highly expressed in the testis and involved in mineralization of tooth enamel (133, 134). ACP6 is mitochondrial, supposed to participate in lipid metabolism through its characterized activity of hydrolyzing lysophosphatidic acid (135). Based on similarity to ACP5, ACP7 is predicted to be a putative tartrate-resistant phosphatase, a member of purple acid phosphatase family of metallophosphoesterase superfamily. The most probable locations of ACP7 are supposed to be extracellular space and cytosol. Studies on substrate specificities of ACP isoenzymes 1–7 are scarce and fragmentary, not enabling a conclusion on their specific catalysis of ThMP hydrolysis. Additionally, the substrate specificity may differ in the isoforms of each gene product, arising due to posttranscriptional (alternative splicing) or posttranslational modifications. Hence, Figure 3 shows all of the ACP isoenzymes expressed in the gut as potential catalysts of ThMP hydrolysis.

A deficiency of acid phosphatase activity in the lysosomal fraction, presumably due to impairment of the ACP2 protein, manifests as intermittent vomiting, hypotonia, lethargy, opisthotonos, terminal bleeding and death in early infancy (136). Many of these symptoms are also present in the thiamine deficiency states (137), supporting involvement of ACP2 in thiamine metabolism. Pathogenic mutation of the tartrate-resistant acid phosphatase ACP5 leads to a strong predisposition to autoimmune diseases, associated with the accumulation of phosphorylated osteopontin, involved in immune regulation and in bone resorption (138). As shown in the next sections, thiamine is tightly linked to immunity. Furthermore, both ACP5 (30) and a ThMP analog benfotiamine (139) are involved with Akt signaling. These lines of evidence favor catalytic activity of ACP5 in ThMP hydrolysis.

Thiamine-specific phosphatases of the bovine brain synaptosomes have been studied by binding them to a thiamine-modified affine sorbent, followed by MS identification of the eluted proteins. Only bacterial paralogues of mammalian proteins with phosphatase activities are identified by the procedure, probably due to poor coverage of the bovine genome. According to the sequence and structural alignment of these identified phosphatases, an apyrase encoded by the ENTPD2 gene, that is expressed in different tissues, including colon and small intestine, may possess the ThDP phosphatase activity in mammals (140). Apyrases, or ectonucleoside diphosphohydrolases, may catalyze hydrolysis of triphosphonucleotides to their monophosphates. Furthermore, these membrane-bound and soluble hydrolases may also act as monophosphatases of the diphosphonucleotides, catalyzing the hydrolysis of ThDP to ThMP (Figure 3).

Thiamine triphosphate is a non-coenzyme derivative of thiamine, probably involved in acetylcholine neurotransmission (141–143). Unlike the substrate-specific enzyme that hydrolyzes ThTP, synthesis of ThTP is currently known to be catalyzed only by the enzymes producing ATP through ADP phosphorylation. That is, ThTP is synthesized by adenylate kinase 1 (AK1) in cytoplasm and by F_1_F_o_-ATP synthase in the mitochondria (140). Mitochondrial ThTP synthesis is tissue-specific and involves an unidentified regulator of F_1_F_o_-ATP synthase linked to pyruvate oxidation (140). Other isoenzymes of AK, shown in Figure 3, are expressed in the gut, having different cellular locations. Their ability to synthesize ThTP and the substrate specificity has not been tested.

The adenylated form of ThTP (AThTP) is found in mammalian tissues, but the enzyme(s) of its synthesis lose their activity during purification, and this interferes with their identification (140). Levels of ThTP and AThTP are higher in fast growing, non-differentiated cells, highlighting the significance of these thiamine derivatives in cellular differentiation (144).

Metabolic transformations and known roles of thiamine and its different natural derivatives are summarized in Table 2.

**Table 2:** Metabolism and roles of thiamine and its derivatives in gastrointestinal system.

Thiamine compound	Role in gastrointestinal system	Chemical transformations in gastrointestinal system
Thiamine	Is transported to enterocytes, directly through the thiamine and OCT transporters, or coupled to ThMP production in transphosphorylation by intestinal alkaline phosphatase (ALPI). May be exchanged for proton through MATE. Important for systemic supply.	Precursor for ThDP biosynthesis by thiamine diphosphokinase (TPK). May be degraded or modified to thiamine antagonists by thiaminases I and II, identified in fish and microbes. Thiamine degradation in mammalian tissues suggests existence of mammalian thiaminases.
ThMP	May be exchanged for folate through SLC19A1. Important for systemic supply.	Hydrolysed in lumen by intestinal alkaline phosphatase (ALPI). Product of thiamine transphosphorylation by intestinal alkaline phosphatase (ALPI).
ThDP	Essential coenzyme of central metabolism. May be exchanged on external folate through SLC19A1 and returned to enterocyte through SLC44A4.	Product of the reactions catalyzed by thiamine diphosphokinase (TPK) or thiamine triphosphatase (ThTPase). May be hydrolysed to ThMP by apyrase(s) (ENTPD). In the reaction with ATP, catalyzed by unidentified enzyme(s), produces regulatory derivative, adenylated ThTP.
ThTP and adenylated ThTP	Specific action in gastrointestinal system is not characterized. In general, these derivatives regulate metabolic enzymes involved in ACh production in mammals through different mechanisms. ThTP regulates rapsyn - scaffolding protein of ACh synapses. ThTP is presumed to be involved into thiamine co-release with ACh at the synapses.	ThTP is synthesized from ThDP by adenylate kinase(s) (KAD) and hydrolysed to ThDP by thiamine triphosphatase (ThTPase). ThTP may be hydrolysed to ThMP by apyrase(s) (ENTPD). In the brain, but not liver, mitochondria ThTP may be formed by ATP synthase in the proton-gradient-dependent manner.

## 5 Transcriptomics of the enterocytes before and after gastric bypass implies increased thiamine demand in humans long term after the bypass

**Figure 4:**
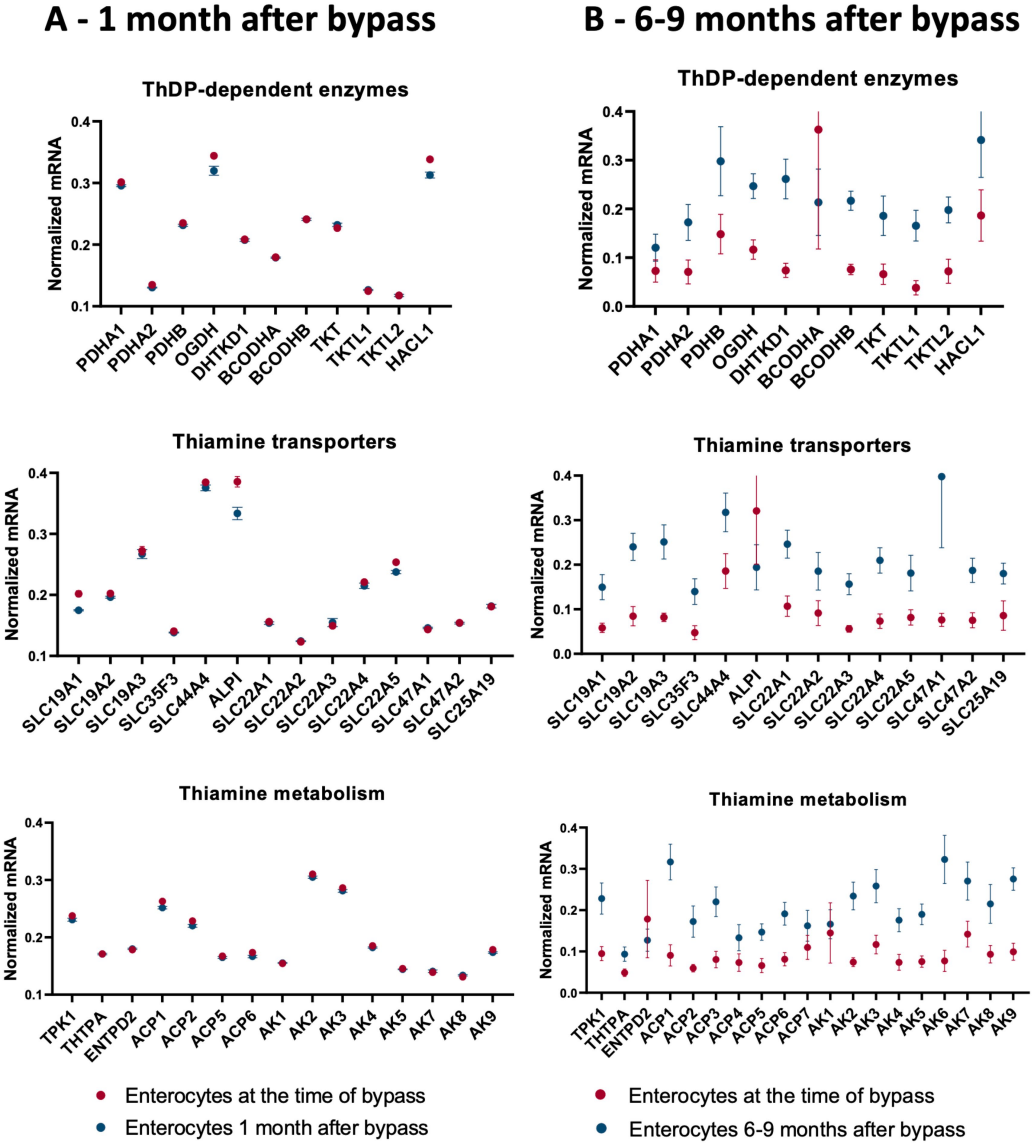
Changes in the averaged levels of mRNA for proteins of thiamine-dependent metabolism after gastric bypass. The relative abundance of mRNAs for the ThDP-dependent enzymes, thiamine transporters and enzymes of thiamine metabolism in jejunum enterocytes before and after gastric bypass is shown. The normalized mRNA signals are calculated as described in the legend to Figure 1. The data are shown as mean ± SEM. **(A)** Experiment GSE113819 {Platform GPL17586 (HTA-2_0) Affymetrix Human Transcriptome Array 2.0 [transcript (gene) version], 5 datasets for enterocytes before bypass: GSM3120595, GSM3120597, GSM3120599, GSM3120601, GSM3120603 - and 5 datasets for enterocytes 1 month after bypass: GSM3120594, GSM3120596, GSM3120598, GSM3120600, GSM3120602}. **(B)** Experiment GSE214758 [Platform GPL20795, HiSeq X Ten (*Homo sapiens*), 9 datasets for enterocytes before bypass: GSM6615629, GSM6615631, GSM6615633, GSM6615637, GSM6615641, GSM6615643, GSM6615645, GSM6615647, GSM6615649 - and 9 datasets for enterocytes 6–9 months after bypass: GSM6615630, GSM6615632, GSM6615638, GSM6615640, GSM6615642, GSM6615644, GSM6615646, GSM6615648, GSM6615650].

**Figure 5:**
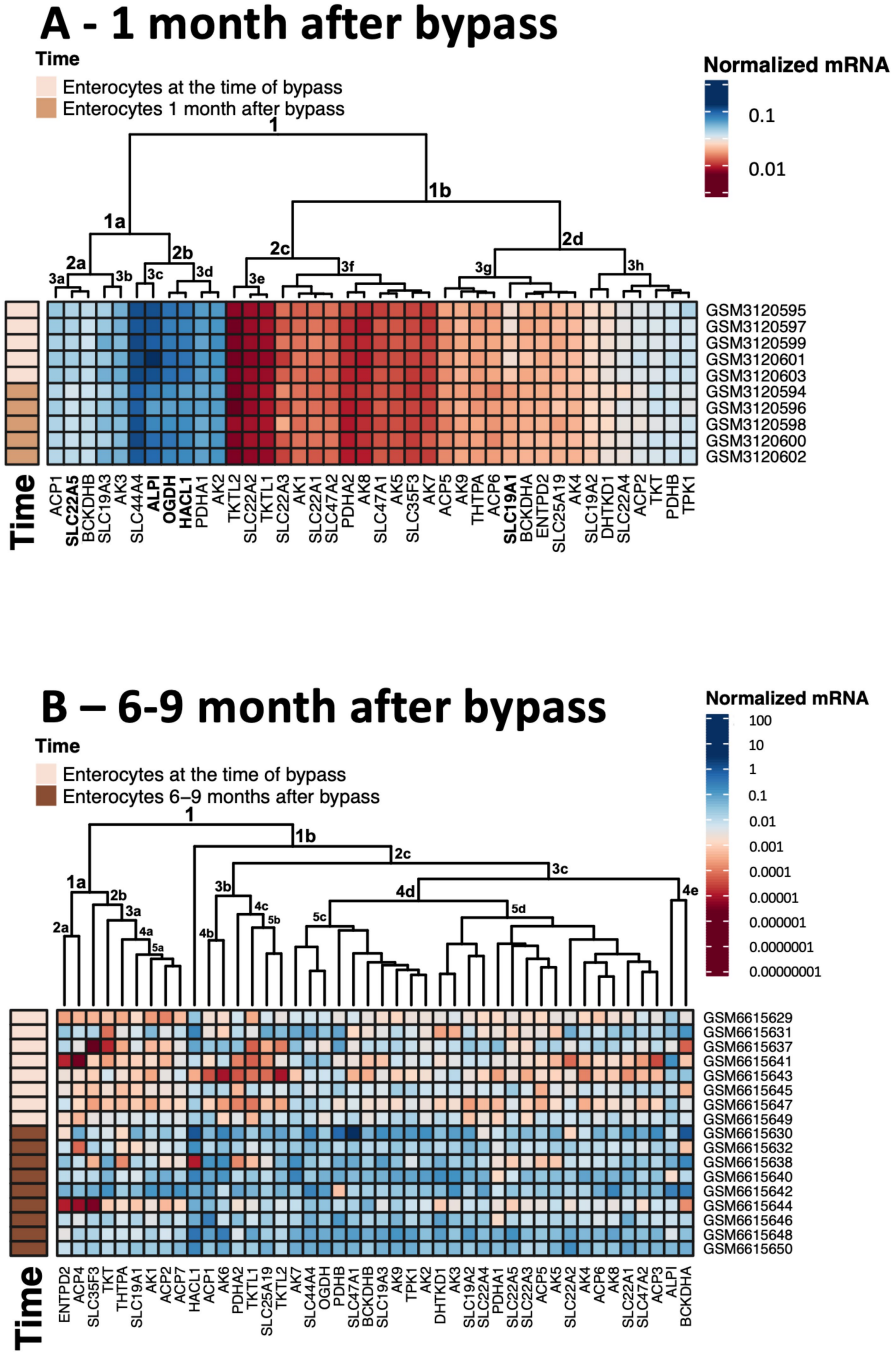
Heatmaps showing transcriptomics changes in the thiamine-dependent metabolism after gastric bypass. The transcriptomics experiments are identified in Figure 4. The normalized mRNA signals for the enzymes of thiamine-dependent metabolism in jejunum enterocytes before and after gastric bypass are shown in decimal logarithm scale according to the color code legend accompanying each heatmap. The heatmaps are produced using R program. **(A)** The normalized mRNA signals before and 1 month after gastric bypass. **(B)** The normalized mRNA signals before and 6–9 months after gastric bypass. The dataset GSM6615633 is excluded, as many transcripts are not identified in this experiment.

Thiamine deficiency often develops after bariatric surgery, including gastric bypass (145–147). Independent published data provide transcriptomics analysis of the enterocytes at one (15–45 days) (148) and 6–9 (115) months after gastric bypass. We have used these data to answer the question of what happens to thiamine-dependent metabolism after gastric bypass. To compare the levels of mRNAs for proteins involved in thiamine-associated metabolism across different experiments, they are normalized to the summarized transcripts of GAPDH+ACTB+TUBA1A, as described previously (149). The normalized levels are shown as averages between the datasets before and after gastric bypass (Figure 4), and in each of the analyzed datasets (Figure 5). As seen from Figures 4, 5, no gross changes are observed in human jejunum enterocytes 1 month after gastric bypass, similar to findings in the mouse model of the bypass, where an initial metabolic perturbation in response to the surgery, manifesting on the 9^th^ day, is reversed to the control state 2 months post-bypass (148). However, a comparison of either the average transcript levels (Figure 4A), or separate datasets of human enterocytes (Figure 5A) reveals minor, but consistent decreases in transcripts for ALPI > SLC19A1 ≈ OGDH ≈ HACL1 > SLC22A5 1 month after the bypass, suggesting a decrease in thiamine-dependent metabolism.

Clusterization of transcript levels groups the transcripts according to their expression level and coincidence of the variations in different datasets (Figure 5). For instance, cluster 1a in Figure 5A comprises proteins whose transcript levels are higher than those of proteins in cluster 1b. Interestingly, each of the clusters comprises one of the well-characterized thiamine transporters: SLC19A3 shares the cluster 1a with thiamine/ThDP-dependent proteins of higher expression, while SLC19A2 shares the cluster 1b with thiamine/ThDP-dependent proteins of lower expression. Overall, the three well-defined clusters 1a, 2c, 2d comprise transcripts of the high, low and intermediary levels of expression, correspondingly. Transcripts of the thiamine/ThDP-dependent proteins which are most obviously decreasing 1 month after the bypass (Figure 4A, gene names in bold in Figure 5A) belong to the proteins with the high (ALPI, OGDH, HACL1, SLC22A5 in cluster 1a) or intermediary (SLC9A1in cluster 2d) expression (Figure 5A). Increasing levels of clusterization (2, 3 etc.) group proteins of increasing transcriptional coincidence, i.e., proteins whose transcript levels show coupled variations across the datasets. For instance, despite the overall similarity between the transcription profiles before and 1 month after the bypass, the transcripts show featured variations in each of the cluster 2a or 2b. In particular, variations in the transcripts for the ThDP-dependent enzymes with relatively high enterocytic expression, i.e., OGDH, HACL1 and PDHA1, are coupled with those in the transcripts for ALPI and ThDP transporter SLC44A4, all belonging to the cluster 2b including the three proteins with decreased transcripts 1 month after the bypass (Figure 5A in bold). The cluster 2a includes proteins with a lower, compared to the cluster 2b, expression, with the decreasing transcriptional level of SLC22A5 1 month after the bypass coupled to the variations of BCKDHB and SLC19A3 transcripts (Figure 5A). Reduced folate/ThDP exchanger SLC19A1, demonstrating decreased transcript levels 1 month after the bypass, shares the cluster (3 g) with mitochondrial ThDP transporter SLC25A19, ThTPase and BCKDHA (Figure 5A).

In contrast to the transcriptomic pattern 1 month after the bypass, human jejunum enterocytes 6–9 months after the bypass exhibit significant perturbations in their transcriptomics profiles (115). In particular, significant changes in the levels of transcripts of genes encoding TCA cycle enzymes and associated proteins are observed (115). In good agreement with this finding, our analysis of the selected transcripts characterizing thiamine-dependent metabolism, shows an overall increase in the transcripts for the related proteins (Figures 4, 5). In particular, up-regulation of the transcripts for the rate-limiting enzyme of the TCA cycle, 2-oxoglutarate dehydrogenase (OGDH), and for the TCA-cycle-affiliated proteins, such as subunits of pyruvate dehydrogenase (PDH) and branched chain 2-oxo acids dehydrogenase (BCODH) is observed. The upregulation of ThDP-dependent DHTKD1 protein 6–9 months after the gastric bypass surgery (Figures 4B, 5B) is similar to the long-term increase of DHTKD1 protein observed after another type of surgical intervention (laminectomy) (48). In accord with the upregulated expression of all the ThDP-dependent dehydrogenases, transcripts of the thiamine/ThDP transporters and the major producer of ThDP from thiamine, i.e., thiamine diphosphokinase (TPK), as well as probable ThMP phosphatase ACP1, also undergo a long-term upregulation (Figure 4B).

Heatmap in Figure 5B shows that the first level of clusterization results in separating the ThDP-dependent TKT in cluster 1a from all the other ThDP-dependent enzymes, combined in cluster 1b. That said, the homooligomeric ThDP-dependent enzymes participating in metabolism of glucose and amino acids, i.e., TKT, OGDH, DHTKD1, preserve their cluster partners in both of the studied experiments. TKT has a common cluster (2d in Figures 1A, 5A in Figure 5B) with ThTPA and SLC19A1; OGDH shares its cluster (1a in Figures 5A,C in Figure 5B) with SLC44A4 and SLC19A3; DHTKD1 keeps associated with SLC19A2 and SLC22A4 (clusters 3 h in Figures 5A,D in Figure 5B). For the heterooligomeric ThDP-dependent enzymes, i.e., PDH and BCOADH, the cluster partners of the subunits *α* and *β* may be switched. For instance, OGDH shares the cluster 3d with PDHA1 in Figure 5A, while in Figure 5B OGDH occupies the same cluster 5c with PDHB, while PDHA1 is moved to the DHTKD1-comprising cluster 5d. BCKDHB partners with ALPI in the cluster 1a in Figure 5A, but the ALPI partner in cluster 4e of Figure 5B is BCKDHA. As a result, a comparison of the two independent transcriptomics experiments reveals stable associations between specific proteins of thiamine/ThDP metabolism and ThDP-dependent enzymes in enterocytes.

The observed upregulation of not only the ThDP-dependent dehydrogenases limiting and feeding the TCA cycle, but also the thiamine transporters and ThDP-producing enzyme TPK1 (Figures 4B, 5B), may compensate for decreased levels and/or increased demands of thiamine in enterocytes 6–9 months after gastric bypass, facilitated by the higher expression of the proteins binding thiamine or ThDP. Indeed, increased activities of ThDP-dependent dehydrogenases (assayed *in vitro*) are known as a compensatory response to their *in vivo* inhibition (38, 150). Cellular exposure to TD strongly stimulates OGDH activity upon the following incubation with ThDP (29). Hence, the upregulation of the thiamine/ThDP-dependent protein transcripts 6–9 months after gastric bypass (Figures 4B, 5B) maybe a biochemical indicator of thiamine insufficiency in the enterocytes. Remarkably, the upregulation is observed after initial decrease in the five thiamine/ThDP-dependent proteins 1 month after the bypass (Figures 4A, 5A).

According to the overall transcriptomics analysis of the long-term (6–9 months) changes in jejunum enterocytes after the bypass (115), there are interactions between the TCA cycle gene cluster and other significantly affected pathways that include genes linked to the cell cycle G2/M DNA damage checkpoint regulation. The DNA damage checkpoint is also involved in cellular repair and differentiation (151). Strong involvement of thiamine transporters in the differentiation of enterocytes is suggested by approximately 6-fold higher thiamine uptake, corresponding to elevated expression of SLC19A2 and SLC19A3, in the differentiated jejunum epithelial cells of the villi, compared to the non-differentiated jejunum epithelial cells of the crypt (152). Positive action of high doses of thiamine under metabolic perturbations including surgeries (48, 115, 149, 153–155) suggest that cellular protection and/or repair requiring cell differentiation, increase demand of thiamine. After surgical perturbations in enterocytic integrity, elevated cellular growth and differentiation, which occur within days, is followed by temporal organ-specific adaptations to the bypass (148). Both phases are characterized by high metabolic demands. If the increased demand is not met by the increased thiamine supply, a state of TD may easily follow, first in enterocytes, and after some period at the level of organism, most often manifested as Wernicke encephalopathy (145–147). Thus, bariatric surgery-induced upregulation of thiamine-dependent metabolism in jejunum (Figures 4B, 5B) may manifest metabolic remodeling addressing insufficient levels of thiamine in post-bypass enterocytes.

## 6 Molecular basis of the thiamine-induced improvements in gastrointestinal disorders

As mentioned above, despite the direct exposure of the gastrointestinal system to nutritional supply, this system often exhibits thiamine-responsive dysfunctions even when no known TD signs are evident in other tissues. The essential role of ThDP in mitochondrial energy production (Figure 1) is universal for all tissues and therefore can hardly explain specific and primary vulnerability of the gastrointestinal system. This energetic role is usually considered in the context of the specific vulnerability to TD of tissues with high energy demand, such as the heart and the brain. These tissues are considered to be the last to decrease their thiamine content during TD and the first to replete it upon thiamine administration, with the liver serving as the thiamine depot for other tissues (156). Nevertheless, if drugs inhibit ThDP-dependent enzymes, as, e.g., omeprazole does (157), direct exposure of the gastrointestinal system to oral drugs may increase its vulnerability compared to other tissues. Based on currently available data, we also suggest several other origins of TD restricted to the gastrointestinal system.

First of all, the dual role of the gastrointestinal system - dedicated not only to nutrient absorption from the lumen, but also to their supply to other tissues via the blood - may present one of the reasons for the intestine-specific susceptibility to TD. That is, the function of the gastrointestinal system requires a balance between thiamine absorption and its supply to other tissues. Mechanisms controlling the balance between the two processes are not well characterized. However, as shown above in the analysis of thiamine/ThDP-dependent metabolism in enterocytes after gastric bypass, increased thiamine demand by other tissues may impair the thiamine status of the gastrointestinal system.

Furthermore, a major difference between the gastrointestinal system and other tissues is the gut microbiome. It is important to note that microbes can synthesize not only thiamine but also thiamine antagonists. This may occur as part of natural metabolism (158) or when microbes are exposed to drugs that undergo aberrant reactions, such as metronidazole (159–161). Microbial enterotoxins alter gene expression in the gut epithelium, leading to enteropathies (162). Proinflammatory cytokines inhibit enterocytic thiamine uptake at the transcriptional level (163). Thiamine transport is also significantly reduced by bacterial lipopolysaccharide and in sepsis (163, 164). Yet another mechanism contributing to the specific vulnerability of the gastrointestinal system to TD may involve the gut-brain axis, which depends on vagal tone and acetylcholine neurotransmission, for which thiamine co-release is known (165–167). In addition to the parasympathetic regulation, acetylcholine is currently suggested to be a paracrine signal of peripheral tissues, particularly in pancreas and epithelial cells (168, 169).

Below, data on molecular mechanisms underlying thiamine-responsive gastrointestinal dysfunctions are considered in more detail.

### 6.1 Exposure of the intestine to bacterial enzymes degrading thiamine or producing thiamine antagonists

Abnormal proliferation of gut bacteria in the upper small intestine, defined by the term ‘small intestinal bacterial overgrowth’ (SIBO), is associated with symptoms such as bloating, abdominal pain, excessive foul-smelling flatulence, constipation and/or diarrhea. The etiology of SIBO is varied and complex, with relapse commonly occurring after conventional treatment. Research suggests that SIBO is involved in up to 78% of irritable bowel syndrome (IBS) cases, the most commonly diagnosed disorder of gastrointestinal motility (170). SIBO has been associated with dysfunctional intestinal motility, characterized by inadequate peristaltic action of gastrointestinal smooth muscle (171–174), hypochlorhydria, and pancreatic exocrine insufficiency (175). As mentioned above, such symptoms are often responsive to thiamine administration. A link between SIBO and TD is provided by the notion that SIBO may increase bacterial transformations of thiamine, which are far more variable than those in mammals. In particular, this concerns the action of bacterial thiaminases I and II, which catalyze biosynthesis of thiamine antagonists or thiamine degradation, respectively. Thiaminase I catalyzes the substitution of thiamine heterocycles with catalytically inactive heterocycles from xenobiotics. For instance, in the presence of the antibiotic metronidazole, thiaminase I substitutes the thiazolium ring of thiamine with the imidazolium ring of metronidazole, resulting in a thiamine antagonist which can inhibit TPK (160). Microbes also synthesize naturally occurring thiamine antagonists, such as 2’-methoxyThDP (158). Thiaminase II degrades thiamine, splitting it into two heterocycles. Immobilized thiaminase II has been applied as an anticancer approach to deplete thiamine in cancer cells (176). Thiaminase II in fern extracts exerts an effect on neurotransmission which, similar to that of the thiamine antagonist pyrithiamine, can be counteracted by the addition of thiamine (177). It may thus be suggested that direct exposure of the gut to the microbiome - capable of synthesizing thiamine antagonists or possessing thiamine-degrading thiaminase II - may first of all affect the gut, leading to gastrointestinal beriberi, especially under conditions of SIBO or other disturbances of the gut microbiome. If these conditions are not treated, TD may spread from the gastrointestinal system to other tissues, as observed in metronidazole-induced encephalopathy (159).

### 6.2 Thiamine supports intestinal barrier integrity

Disturbed intestinal barrier function has been increasingly studied in recent years and is now widely recognized as a prominent feature of many chronic diseases, pertaining not only to the gut (178), but also to neurological, psychiatric, cardiovascular, and autoimmune conditions. Increased intestinal permeability can facilitate the entry of food antigens, bacteria and bacterial components into systemic circulation, which are thought to provoke systemic inflammatory responses. The intestinal epithelial barrier shares several key morphological and functional characteristics with the blood–brain barrier. The tight junctions of both structures are composed of transmembrane proteins such as claudin, occludin, zonula occluden (ZO), and endothelial cell-selective adhesion molecules. Thiamine is necessary for maintaining intestinal epithelial cell bioenergetics, and reduced activity of thiamine-dependent enzymes may lead to a defective gut barrier (34). TD is known to disrupt the blood brain barrier, featuring loss of occludin, ZO-1 and ZO-2 (179, 180). It is therefore possible that similar mechanisms could be at play in the gastrointestinal tract. Notably, TD leads to disturbed expression of junction protein subtypes in *Ctenopharyngodon idella* (181). Moreover, thiamine administration enhances claudin-1, claudin-4, ZO-1, and occludin in ruminal epithelium (22). Thiamine facilitates the protective action of secretory IgA against immunogenic threats in enterocytes (182). The prevention of intestinal barrier dysfunction by secretory IgA, along with its immunomodulatory properties, may play a role in IBD, including ulcerative colitis and Crohn’s disease (183). Poor thiamine status is frequently reported in IBD, mostly assumed to be a consequence of malabsorption (184–187). TD aggravates ulcerative colitis in mice, associated with the promoted infiltration of proinflammatory M1 macrophages into colonic lamina propria (188). The underlying mechanism is TD-induced impairment of PDHC activity, which causes remodeling of glucose metabolism in the macrophages.

Other mechanisms may also contribute to IBD, too. In particular, IBD is associated with hypoxia in enterocytes (189). Hypoxia has recently been shown to downregulate thiamine transporters in a colonic cell line, impairing the thiamine uptake that results in a localized intracellular deficiency (190). Alkaline phosphatase of the brush border of the intestines, which participates in thiamine transport (Figure 2), plays protective roles against pathogenic infection (191) and bacterial toxins (LPS) in the gut, and may counteract inflammation (192).

A combination of exogenous and endogenous factors is known to influence intestinal barrier function, including both acute and/or chronic immune dysregulation (193). TD may induce negative developments in the gastrointestinal system, which can be counteracted by thiamine supplementation. By normalizing or increasing the efficiency of glucose metabolism, thiamine decreases the degradation of amino acids as energy substrates and increases protein synthesis, which is essential for maintaining tight junctions and intestinal barrier integrity (194). Remarkably, TD in rats causes a 42–66% reduction of brush border enzyme activities and a 20% reduction in intestinal weight with significant thinning of the microvillus membrane (195). This may be the result of disturbed function of the ThDP-dependent 2-oxoglutarate dehydrogenase (Figure 1), as a universal effect of its inhibition is perturbation in relative amino acid abundance and a subsequent decrease in protein synthesis (196, 197). In turn, villous atrophy can impair nutrient absorption and is one of the primary mechanisms underpinning extensive nutritional deficiencies found in Celiac and inflammatory bowel diseases (198). Furthermore, atrophy and inflammation of mucosal surfaces in the gut are documented in experimental TD (82), and high doses of thiamine have been trialed in two studies showing that thiamine reduced fatigue associated with IBD (199, 200).

### 6.3 Thiamine and intestinal inflammation

Thiamine is considered a natural anti-inflammatory compound (201). Studies in both goats (202) and cows (203) indicate that thiamine has anti-inflammatory effects on the ruminal epithelium, ameliorating in particular the intestinal inflammation and barrier permeability caused by high-concentrate diet (27). As considered in Section 3 above, thiamine affects the composition of symbiotic microbiota, and ThDP-dependent bacterial butyrate synthesis in particular. Knockouts of the ThDP transporter *SLC44A4* in mice display an upregulation of genes associated with colonic inflammation, and increased susceptibility to dextran sodium sulfate-induced colitis, accompanied by significant weight loss and shortening of the colon (204).

A number of positive anti-inflammatory actions of thiamine include activation of Nrf2 and decreased ROS levels, enhanced activity of mitochondrial respiratory chain complexes I-IV, downregulation of endoplasmic reticulum stress, and suppression of gene expression associated with mitophagy, oxidative stress, and proinflammatory cytokines (205). Remarkably, the antioxidant effects of thiamine, including Nrf2 activation, are observed even when the thiamine disulfide forms— which should decrease cellular redox potential— are administered (206). These pharmacological forms, such as sulbutiamine or TTFD, may penetrate cell membrane better than thiamine. Inside the cells, they are reduced, particularly by the thioredoxin and glutaredoxin system, simultaneously stimulating antioxidant defense through Nrf2 activation (206). One may suggest that Nrf2 activation is specific to the disulfide forms of thiamine, not inherent in thiamine itself, as only the thiamine disulfides undergo intracellular reduction. The associated shift in the cellular redox state may cause the activation of Nrf2, leading to this additional positive effect on metabolism of high doses of thiamine disulfides, compared to thiamine. The Nrf2 activation along with the thiamine formation may explain why high doses of the thiamine disulfides, expected to decrease cellular redox potential, do not have any negative action even upon the long-term administration (155).

By maintaining integrity of the intestinal barrier via complex mechanisms, the vagus nerve and its major neurotransmitter, acetylcholine (ACh), play a crucial role in coordinating adaptive neural and endocrine responses of the gastrointestinal system, including those against infection and inflammation (207). SIBO and intestinal hypomotility are highly prevalent in patients with anti-ACh receptor antibodies (208, 209).

### 6.4 Acetylcholine-dependent mechanisms of thiamine action in gastrointestinal disorders

Many independent data associate gastrointestinal disorders with perturbed ACh signaling. ACh exerts tonic effects to maintain constriction of the lower esophageal sphincter, with ACh signaling compromised in a rat model of gastrointestinal reflux disease (210). Drugs which block the action of ACh can cause abnormal relaxation of the sphincter and reflux (211). The anti-cholinergic agents atropine and papaverine induce constipation in animals (23). In contrast, preserving ACh levels through acetylcholinesterase inhibitors improves gastrointestinal reflux disease (212). Pro-cholinergic pharmacological agents also provide symptomatic improvement in FGIDs (213, 214). Pharmacological forms of thiamine exhibit interactions with the ACh-dependent effects. That is, TTFD prevents the gut paralysis induced by atropine or papaverine (23), while sulbutiamine promotes cholinergic neurotransmission, potentiating the action of ACh esterase inhibitors (215). Interestingly, structure of an inhibitor of ACh esterase, acotiamide (brand name acofide), approved in Japan as a prokinetic motility drug against functional dyspepsia (216–218), combines the two heterocycles, one of them thiazole, that may be considered as structural mimics of the thiazolium and aminopyrimidine heterocycles of thiamine.

An intimate relationship between thiamine and cholinergic neurotransmission relies on both the coenzyme and non-coenzyme actions of thiamine and its derivatives. In cholinergic cells, the ThDP-dependent pyruvate dehydrogenase complex synthesizes the ACh precursor acetyl-CoA (Figure 1) (219, 220). Distribution of this acetyl-CoA between the oxidation in the TCA cycle and participation in ACh synthesis is regulated through limitation of the TCA cycle rate by the ThDP-dependent OGDH, and the non-coenzyme action of thiamine and derivatives on the other enzymes involved (221). TD-perturbed function of ThDP-dependent dehydrogenases of TCA cycle (Figure 1) is a well-known contributor to impaired synthesis of ACh (219). The ensuing mitochondrial dysfunction of cholinergic cells increases their susceptibility to different insults (222–224). In particular, neurons of the gastrointestinal system are highly sensitive to oxidative stress (219, 225, 226), which is a general hallmark of TD (227). Cytosolic oxidative stress can inactivate nicotinic ACh receptors in neurons, decreasing ACh-evoked currents (228). Oxidative stress is considered to contribute to the pathophysiology of IBD (229, 230).

Independent of metabolic action as the coenzyme ThDP, thiamine is essential for axonal membrane excitability, playing a significant role in development of the action potential (231). This non-coenzyme action of thiamine in neuronal signaling is further supported by the identified molecular targets of thiamine and its non-coenzyme derivative ThTP. ThTP-dependent phosphorylation of rapsyn regulates ACh neurotransmission, as rapsyn is a scaffolding protein of the post-synaptic membrane of neuromuscular junctions, specifically associated with nicotinic ACh receptors (232). Hydrolysis of ThTP by synaptic membrane-bound protein(s) different from the well-characterized soluble ThTPase of cytosol, is supposed to be involved with synaptic function (233, 234). Thiamine binds to a bitter taste receptor, which modifies ileum contraction (235) and provokes ACh-induced contraction of jejunum (236). At a high concentration (0.05 mM), thiamine also binds to an isolated nicotinic ACh receptor (237). Probably, the low binding affinity is an artifact of the receptor isolation, as the physiologically relevant affinity of thiamine to the receptor may require protein–protein interaction within the native structure of the synapses. The non-coenzyme action of thiamine in neurotransmission is further supported by the action of the thiamine analog oxythiamine, whose diphosphorylated derivative (oxyThDP), formed by TPK *in vivo*, is an antagonist of the coenzyme action of thiamine. As a result, oxyThDP inhibits ThDP-dependent dehydrogenases, whose function is required for ACh synthesis, particularly pyruvate dehydrogenase complex generating ACh precursor acetyl-CoA (Figure 1). However, in superfused rat brain slices, oxythiamin enhances the release of synaptic acetylcholine, thus mimicking the non-coenzyme action of thiamine in facilitating ACh neurotransmission (238).

ACh is a major neurotransmitter of vagus nerve, coordinating the brain-gut axes (239). Vagus nerve stimulation can lead to gastrointestinal improvements (240), e.g., can increase gastric emptying through acting on the pyloric sphincter (241) and may be a treatment for gastroparesis (242). Potentiation of ACh release through transcutaneous vagal neuromodulation is known to enhance gut motility, reduce inflammation by suppressing TNF-*α*, and preserve epithelial tight junction integrity through the activation of enteric glial cells (243). The data indicate that TD-perturbed ACh synthesis and neurotransmission in vagus nerve may induce gastrointestinal dysfunction. However, in this case ACh synthesis and signaling are controlled by neurons of central nervous system. As discussed above, sufficient levels of thiamine in the brain are supported at the expense of other tissues, causing TD in these other tissues long before the TD occurs in the brain. Hence, when TD is limited to the gastrointestinal system, the pathology-relevant perturbation of ACh signaling may rather be expected in the enteric nervous system (ENS). ENS may regulate gastrointestinal function both with and without the input of central nervous system (244–246). In particular, the independent function of ENS is manifested in the intestinal peristaltic reflex.

ACh is a primary neurotransmitter of different types of ENS neurons, i.e., intrinsic primary afferent neurons, excitatory motor neurons, and interneurons (245). The thiamine-induced increases in contractions of the intestine smooth muscle and peristalsis, observed in a number of independent *in vitro* experiments on muscle strips of gastrointestinal tract (207, 219–222), support the action of thiamine on ACh signaling in ENS in the absence of central regulation (236). Synaptic co-release of thiamine and ACh, with thiamine facilitating the neurotransmission through its non-coenzyme action, is known from the early works of Minz and von Muralt on neuromuscular junctions (143, 165–167). In accordance with these observations, addition of thiamine and ThMP modulate synaptic transmission, electric and contractile activity of the smooth muscle strips from gastrointestinal tract (247). Thiamine hydrochloride, thiamine nitrate, thiamine propyldisulfide, TTFD, and some other experimental derivatives increase peristalsis of isolated sections of small intestine from rat (248). In isolated sections of murine jejunum and ileum, thiamine regulates parameters of the ACh-induced contraction in the concentration-dependent manner (236). In isolated duodenal and jejunal segments from cats, dogs, rabbits and guinea pigs the thiamine disulfide derivative TTFD exerts an excitatory effect on motility; sensitivity of the effect to an antagonist of muscarinic acetylcholine receptor atropine suggests involvement of the ACh neurotransmission (249). All forms of thiamine including thiamine hydrochloride, S-benzoyl thiamine disulfide, TTFD, increase contractions of isolated segments of small intestine (248).

In human patients with chronic enterocolitis and colitis, thiamine-induced increases in motor activity of stomach, small and large intestine are detected by electrogastrography and balloon-kymography, with no similar effects of vitamins B6, B12 and C (250). In animal models, TTFD can stimulate intestinal peristalsis within a few minutes after administration, also when mesenteric nerves are cut (251, 252). Intravenous administration of TTFD induces a slight increase in tone and a remarkable increase in the amplitude of rhythmic contractions in the jejunal loop of both non-anesthetized and anesthetized dogs for 20 min (253). TTFD applied topically inside the lumen of the intestine can also elicit such excitation effect (249). Nevertheless, in some studies on TTFD, percent weight gain was lower in the TTFD group (254). Two possible explanations for this effect are proposed: (1) reduced food intake due to an irritant effect of the treatment on the gastrointestinal tract (255, 256) or (2) a stimulant effect of TTFD on metabolism via enhanced noradrenaline secretion and thermogenesis (257).

ACh signaling in non-neuronal cells, employing the nicotinic and muscarinic ACh receptors, is comparable to ACh neurotransmission (168, 169). Therefore, ACh signaling in non-neuronal cells is another process potentially affected by TD before the deficiency develops into the well-known dry or wet beriberi. Glial cells of ENS are stimulated by ACh (245). In mammalian intestine, ACh regulates sodium currents in the apical and basolateral membranes (168). In human pancreas, ACh synthesized by alpha-cells is suggested to activate beta-cells (169). Other *in vivo* actions of ACh in pancreas may be linked to its high thiamine content (89). As considered in Section 3, TD in pancreas strongly affects gastrointestinal function. Perturbed ACh signaling by pancreatic cells under TD may contribute to gastrointestinal dysfunction in addition to insufficient pancreatic synthesis of the enzymes needed for digestion.

Thus, cholinergic neurotransmission and ACh-dependent regulation of non-neuronal cells employ ThDP coenzyme function for ACh synthesis and non-coenzyme action of thiamine in ACh signaling. Both may be impaired by TD, contributing to gastrointestinal dysfunction.

## 7 Conclusion

Various lines of evidence at the molecular level and in model systems provide a mechanistic basis for a number of observations at the physiological level, where thiamine supplementation alleviates gastrointestinal dysfunction, suggesting that insufficient levels of thiamine may underlie this dysfunction. The specific vulnerability of the gastrointestinal system to TD, in the absence of more common signs of wet and dry beriberi, may arise from direct exposure of this system to the gut microbiome and oral drugs, along with the continuous need to redistribute thiamine to other tissues. Cholinergic neurons of the enteric nervous system, which interact closely with gastrointestinal epithelial cells, as well as ACh signaling in non-neuronal cells such as enteric glia and pancreatic cells, depend on the essential role of thiamine in ACh production and the facilitation of ACh signaling. These mechanisms collectively contribute to the unique vulnerability of the gastrointestinal system to TD, even in the absence of classic forms of TD such as dry and wet beriberi. The multitude of proteins involved in mammalian metabolism and function of thiamine is not fully characterized. However, available data point to a much greater complexity of thiamine metabolism and its physiological significance, as commonly considered. This complexity must still be addressed by the identification of all genes involved. Individual differences in genetic variants of the thiamine-dependent proteins, in combination with environmental factors, may underlie personal vulnerabilities to thiamine deficiency, manifesting in an ever-increasing variety of clinical presentations.

## References

[1] Wang Y Huang Y Chase RC Li T Ramai D Li S. Global burden of digestive diseases: A systematic analysis of the global burden of diseases study, 1990 to 2019. Gastroenterology. (2023) 165:773–783.e15. doi: 10.1053/j.gastro.2023.05.050, PMID: 37302558

[2] Peery AF Crockett SD Murphy CC Jensen ET Kim HP Egberg MD. Burden and cost of gastrointestinal, liver, and pancreatic diseases in the United States: update 2021. Gastroenterology. (2022) 162:621–44. doi: 10.1053/j.gastro.2021.10.017, PMID: 34678215 PMC10756322

[3] Huang Z Li Y Park H Ho M Bhardwaj K Sugimura N. Unveiling and harnessing the human gut microbiome in the rising burden of non-communicable diseases during urbanization. Gut Microbes. (2023) 15:2237645. doi: 10.1080/19490976.2023.2237645, PMID: 37498052 PMC10376922

[4] Rome IV. *Criteria*. Rome Found Available at: https://theromefoundation.org/rome-iv/rome-iv-criteria/ (Accessed October 31, 2024).

[5] Drossman DA. Functional gastrointestinal disorders: history, pathophysiology, clinical features, and Rome IV. Gastroenterology. (2016) 150:1262–1279.e2. doi: 10.1053/j.gastro.2016.02.032, PMID: 27144617

[6] Fikree A Byrne P. Management of functional gastrointestinal disorders. Clin Med. (2021) 21:44–52. doi: 10.7861/clinmed.2020-0980, PMID: 33479067 PMC7850201

[7] Sperber AD Bangdiwala SI Drossman DA Ghoshal UC Simren M Tack J. Worldwide prevalence and burden of functional gastrointestinal disorders, results of Rome foundation global study. Gastroenterology. (2021) 160:99–114.e3. doi: 10.1053/j.gastro.2020.04.014 32294476

[8] Grover M Farrugia G Stanghellini V. Gastroparesis: a turning point in understanding and treatment. Gut. (2019) 68:2238–50. doi: 10.1136/gutjnl-2019-318712, PMID: 31563877 PMC6874806

[9] Mazzone A Strege PR Gibbons SJ Alcaino C Joshi V Haak AJ. microRNA overexpression in slow transit constipation leads to reduced NaV1.5 current and altered smooth muscle contractility. Gut. (2020) 69:868–76. doi: 10.1136/gutjnl-2019-318747, PMID: 31757880 PMC7147984

[10] Singh R Wei L Ghoshal UC. Micro-organic basis of functional gastrointestinal (GI) disorders: role of microRNAs in GI pacemaking cells. Indian J Gastroenterol. (2021) 40:102–10. doi: 10.1007/s12664-021-01159-7, PMID: 33738768

[11] Cipriani G Gibbons SJ Kashyap PC Farrugia G. Intrinsic gastrointestinal macrophages: their phenotype and role in gastrointestinal motility. Cell Mol Gastroenterol Hepatol. (2016) 2:120–130.e1. doi: 10.1016/j.jcmgh.2016.01.003, PMID: 27047989 PMC4817106

[12] Wei L Singh R Ha SE Martin AM Jones LA Jin B. Serotonin deficiency is associated with delayed gastric emptying. Gastroenterology. (2021) 160:2451–2466.e19. doi: 10.1053/j.gastro.2021.02.060, PMID: 33662386 PMC8532026

[13] Drossman DA Tack J Ford AC Szigethy E Törnblom H Oudenhove LV. Neuromodulators for functional gastrointestinal disorders (disorders of gut−brain interaction): A Rome foundation working team report. Gastroenterology. (2018) 154:1140–1171.e1. doi: 10.1053/j.gastro.2017.11.279, PMID: 29274869

[14] Bunik VI. A challenging interplay between basic research, technologies and medical education to provide therapies based on disease mechanisms. Front Med. (2024) 11:672. doi: 10.3389/fmed.2024.1464672, PMID: 39228799 PMC11368752

[15] Dalayeli N Hajhashemi V Talebi A Minaiyan M. Investigating the impact of selected B vitamins (B1, B2, B6, and B12) on acute colitis induced experimentally in rats. Int J Prev Med. (2024) 15:61. doi: 10.4103/ijpvm.ijpvm_232_23, PMID: 39742123 PMC11687687

[16] Hua-an X Mei-lin H Li-ping C Tai-zhen L Qi-mei H Hui-xian L. Comparative study of two methods on promoting the passage of gas by anus on patient after section of gynecology. Clin Med Eng. (2009) 16:63–4.

[17] Tjong E Peng Y-Y. Gastrointestinal beriberi and Wernicke’s encephalopathy triggered by one session of heavy drinking. Case Rep Neurol. (2019) 11:124–31. doi: 10.1159/000499601, PMID: 31543793 PMC6739701

[18] Shah HN Bal BS Finelli FC Koch TR. Constipation in patients with thiamine deficiency after roux-en-Y gastric bypass surgery. Digestion. (2013) 88:119–24. doi: 10.1159/000353245 23970020

[19] Shah IA Kawoos Y Shah AI Rabyang S Naqash H Mir MR. Gastrointestinal beriberi as a prodrome of non-alcoholic Wernicke’s encephalopathy: a study of an emerging nutritional deficiency disorder from Kashmir, India. Int J Res Med Sci. (2019) 7:1494–9. doi: 10.18203/2320-6012.ijrms20191496

[20] Fang-Fang Z Zhi-Rong F Yong-Yu X. Effect of vitamin B1 integrated with recovery exercises on the postoperative recovery of gastrointestinal function of patients with transabdominal hysterectomy. J Qilu Nurs. (2012) 1:11.

[21] Ma Y Wang C Elmhadi M Zhang H Liu F Gao X. Dietary supplementation of thiamine enhances colonic integrity and modulates mucosal inflammation injury in goats challenged by lipopolysaccharide and low pH. Br J Nutr. (2022) 128:2147–57. doi: 10.1017/S0007114522000174, PMID: 35057872

[22] Ma Y Zhang Y Elmhadi M Zhang H Wang H. Thiamine alleviates high-concentrate-diet-induced oxidative stress, apoptosis, and protects the rumen epithelial barrier function in goats. Front Vet Sci. (2021) 8:663698. doi: 10.3389/fvets.2021.663698, PMID: 34095275 PMC8173046

[23] Nagata M Sugimoto J. The influences of drugs on the experimental constipation of mice treated with atropine and Papaverine. J Kansai Med Univ. (1973) 25:300–21. doi: 10.5361/jkmu1956.25.3_300

[24] Bunik VI Tylicki A Lukashev NV. Thiamin diphosphate-dependent enzymes: from enzymology to metabolic regulation, drug design and disease models. FEBS J. (2013) 280:6412–42. doi: 10.1111/febs.12512, PMID: 24004353

[25] Ramamoorthy K Sabui S Kim G Fleckenstein JM Sheikh A Said HM. IQGAP-2: A novel interacting partner for the human colonic thiamin pyrophosphate transporter (hcTPPT). Am J Physiol Cell Physiol. (2024) 327:C1451–61. doi: 10.1152/ajpcell.00484.2024, PMID: 39401425 PMC11684876

[26] Patnni C Reggiani C Laforenza U Rindi G. Blood–brain transport of thiamine monophosphate in the rat: A kinetic study in vivo. J Neurochem. (1988) 50:90–3. doi: 10.1111/j.1471-4159.1988.tb13234.x 3335853

[27] Ma J Shah AM Wang Z Fan X. Potential protective effects of thiamine supplementation on the ruminal epithelium damage during subacute ruminal acidosis. Anim Sci J. (2021) 92:e13579. doi: 10.1111/asj.13579, PMID: 34173303

[28] Sangha V Hoque MT Henderson JT Bendayan R. Novel localization of folate transport systems in the murine central nervous system. Fluids Barriers CNS. (2022) 19:92. doi: 10.1186/s12987-022-00391-3, PMID: 36419095 PMC9686069

[29] Bunik VI Aleshin VA Zhou X Tabakov VY Karlsson A. Activation of mitochondrial 2-Oxoglutarate dehydrogenase by Cocarboxylase in human lung adenocarcinoma cells A549 is p53/p21-dependent and impairs cellular redox state, mimicking the cisplatin action. Int J Mol Sci. (2020) 21:3759. doi: 10.3390/ijms21113759, PMID: 32466567 PMC7312097

[30] Lång P Patlaka C Andersson G. Tartrate-resistant acid phosphatase type 5/ACP5 promotes cell cycle entry of 3T3-L1 preadipocytes by increasing IGF-1/Akt signaling. FEBS Lett. (2021) 595:2616–27. doi: 10.1002/1873-3468.14184, PMID: 34418080

[31] Smith TJ Johnson CR Koshy R Hess SY Qureshi UA Mynak ML. Thiamine deficiency disorders: a clinical perspective. Ann N Y Acad Sci. (2021) 1498:9–28. doi: 10.1111/nyas.14536, PMID: 33305487 PMC8451766

[32] Muley A Fernandez R Green H Muley P. Effect of thiamine supplementation on glycaemic outcomes in adults with type 2 diabetes: a systematic review and meta-analysis. BMJ Open. (2022) 12:e059834. doi: 10.1136/bmjopen-2021-059834, PMID: 36008064 PMC9422810

[33] Mori K Naganuma T Kihara A. Role of 2-hydroxy acyl-CoA lyase HACL2 in odd-chain fatty acid production via α-oxidation in vivo. Mol Biol Cell. (2023) 34:ar85. doi: 10.1091/mbc.E23-02-0042, PMID: 37285239 PMC10398889

[34] Tian N Hu L Lu Y Tong L Feng M Liu Q. TKT maintains intestinal ATP production and inhibits apoptosis-induced colitis. Cell Death Dis. (2021) 12:853–11. doi: 10.1038/s41419-021-04142-4, PMID: 34535624 PMC8448773

[35] Van Der FLG Clevers H. Stem cells, self-renewal, and differentiation in the intestinal epithelium. Annu Rev Physiol. (2009) 71:241–60. doi: 10.1146/annurev.physiol.010908.163145, PMID: 18808327

[36] Bunik V Kaehne T Degtyarev D Shcherbakova T Reiser G. Novel isoenzyme of 2-oxoglutarate dehydrogenase is identified in brain, but not in heart. FEBS J. (2008) 275:4990–5006. doi: 10.1111/j.1742-4658.2008.06632.x 18783430

[37] Artiukhov AV Grabarska A Gumbarewicz E Aleshin VA Kähne T Obata T. Synthetic analogues of 2-oxo acids discriminate metabolic contribution of the 2-oxoglutarate and 2-oxoadipate dehydrogenases in mammalian cells and tissues. Sci Rep. (2020) 10:1886. doi: 10.1038/s41598-020-58701-4, PMID: 32024885 PMC7002488

[38] Bunik VI Artiukhov AV Kazantsev AV Aleshin VA Boyko AI Ksenofontov AL. Administration of Phosphonate Inhibitors of dehydrogenases of 2-Oxoglutarate and 2-Oxoadipate to rats elicits target-specific metabolic and physiological responses. Front Chem. (2022) 10:284. doi: 10.3389/fchem.2022.892284, PMID: 35795216 PMC9252169

[39] Sherrill JD Kc K Wang X Wen T Chamberlin A Stucke EM. Whole-exome sequencing uncovers oxidoreductases DHTKD1 and OGDHL as linkers between mitochondrial dysfunction and eosinophilic esophagitis. JCI Insight. (2018) 3:922. doi: 10.1172/jci.insight.99922, PMID: 29669943 PMC5931135

[40] Denton RM Pullen TJ Armstrong CT Heesom KJ Rutter GA. Calcium-insensitive splice variants of mammalian E1 subunit of 2-oxoglutarate dehydrogenase complex with tissue-specific patterns of expression. Biochem J. (2016) 473:1165–78. doi: 10.1042/BCJ20160135, PMID: 26936970 PMC6101200

[41] Tsepkova PM Artiukhov AV Boyko AI Aleshin VA Mkrtchyan GV Zvyagintseva MA. Thiamine induces Long-term changes in amino acid profiles and activities of 2-Oxoglutarate and 2-Oxoadipate dehydrogenases in rat brain. Biochem Mosc. (2017) 82:723–36. doi: 10.1134/S0006297917060098, PMID: 28601082

[42] Shang S Liu J Hua F. Protein acylation: mechanisms, biological functions and therapeutic targets. Signal Transduct Target Ther. (2022) 7:396–30. doi: 10.1038/s41392-022-01245-y, PMID: 36577755 PMC9797573

[43] Fernandes MF Vinolo MAR. Histone acylations as a mechanism for regulation of intestinal epithelial cells. Dig Med Res. (2024) 7:4. doi: 10.21037/dmr-23-3, PMID: 39399394 PMC11469631

[44] Sutendra G Kinnaird A Dromparis P Paulin R Stenson TH Haromy A. A nuclear pyruvate dehydrogenase complex is important for the generation of acetyl-CoA and histone acetylation. Cell. (2014) 158:84–97. doi: 10.1016/j.cell.2014.04.046, PMID: 24995980

[45] Wang Y Guo YR Liu K Yin Z Liu R Xia Y. KAT2A coupled with the α-KGDH complex acts as a histone H3 succinyltransferase. Nature. (2017) 552:273–7. doi: 10.1038/nature25003, PMID: 29211711 PMC5841452

[46] Li W Long Q Wu H Zhou Y Duan L Yuan H. Nuclear localization of mitochondrial TCA cycle enzymes modulates pluripotency via histone acetylation. Nat Commun. (2022) 13:7414. doi: 10.1038/s41467-022-35199-0, PMID: 36460681 PMC9718843

[47] Bunik VI Degtyarev D. Structure-function relationships in the 2-oxo acid dehydrogenase family: substrate-specific signatures and functional predictions for the 2-oxoglutarate dehydrogenase-like proteins. Proteins. (2008) 71:874–90. doi: 10.1002/prot.21766, PMID: 18004749

[48] Boyko AI Karlina IS Zavileyskiy LG Aleshin VA Artiukhov AV Kaehne T. Delayed impact of 2-Oxoadipate dehydrogenase inhibition on the rat brain metabolism is linked to protein Glutarylation. Front Med. (2022) 9:263. doi: 10.3389/fmed.2022.896263, PMID: 35721081 PMC9198357

[49] Aleshin VA Sibiryakina DA Kazantsev AV Graf AV Bunik VI. Acylation of the rat brain proteins is affected by the inhibition of pyruvate dehydrogenase in vivo. Biochem Mosc. (2023) 88:105–18. doi: 10.1134/S0006297923010091, PMID: 37068879

[50] Hansen GE Gibson GE. The α-Ketoglutarate dehydrogenase complex as a hub of plasticity in neurodegeneration and regeneration. Int J Mol Sci. (2022) 23:12403. doi: 10.3390/ijms232012403, PMID: 36293260 PMC9603878

[51] Bunik VI Raddatz G Strumilo S. Translating enzymology into metabolic regulation: the case of the 2- Oxoglutarate dehydrogenase multienzyme complex. Curr Chem Biol. 7:74–93. doi: 10.2174/2212796811307010008

[52] Bunik VI. Redox-driven signaling: 2-Oxo acid dehydrogenase complexes as sensors and transmitters of metabolic imbalance. Antioxid Redox Signal. (2019) 30:1911–47. doi: 10.1089/ars.2017.7311, PMID: 30187773

[53] Goodwin DW. The Wernicke-Korsakoff syndrome: A clinical and pathological study of 245 patients, 82 with post-mortem examinations. JAMA. (1972) 219:389. doi: 10.1001/jama.1972.03190290075032 5162155

[54] Oudman E Wijnia JW Bidesie JR van Dam MJ Oey MJ Smits S. Pediatric Wernicke encephalopathy: A systematic review. Pediatr Rep. (2025) 17:15. doi: 10.3390/pediatric17010015, PMID: 39997622 PMC11859456

[55] Gu Q Li P Gu Q Li P. Biosynthesis of vitamins by probiotic Bacteria In: Rao V Rao L, editors. Probiotics and prebiotics in human nutrition and health. London: IntechOpen (2016)

[56] Wan Z Zheng J Zhu Z Sang L Zhu J Luo S. Intermediate role of gut microbiota in vitamin B nutrition and its influences on human health. Front Nutr. (2022) 9:1502. doi: 10.3389/fnut.2022.1031502, PMID: 36583209 PMC9792504

[57] Arumugam M Raes J Pelletier E Le Paslier D Yamada T Mende DR. Enterotypes of the human gut microbiome. Nature. (2011) 473:174–80. doi: 10.1038/nature09944, PMID: 21508958 PMC3728647

[58] Costliow ZA Degnan PH. Thiamine acquisition strategies impact metabolism and competition in the gut microbe *Bacteroides thetaiotaomicron*. mSystems. (2017) 2:e00116–7. doi: 10.1128/mSystems.00116-17, PMID: 28951891 PMC5613172

[59] Soto-Martin EC Warnke I Farquharson FM Christodoulou M Horgan G Derrien M. Vitamin biosynthesis by human gut butyrate-producing Bacteria and cross-feeding in synthetic microbial communities. MBio. (2020) 11:e00886–20. doi: 10.1128/mBio.00886-20, PMID: 32665271 PMC7360928

[60] Magnúsdóttir S Ravcheev D de Crécy-Lagard V Thiele I. Systematic genome assessment of B-vitamin biosynthesis suggests co-operation among gut microbes. Front Genet. (2015) 6:148. doi: 10.3389/fgene.2015.00148, PMID: 25941533 PMC4403557

[61] Park J Hosomi K Kawashima H Chen Y-A Mohsen A Ohno H. Dietary vitamin B1 intake influences gut microbial community and the consequent production of short-chain fatty acids. Nutrients. (2022) 14:2078. doi: 10.3390/nu14102078, PMID: 35631219 PMC9147846

[62] Bermúdez-Sánchez S Bager P Dahlerup JF Baunwall SMD Licht TR Mortensen MS. Thiamine-reduced fatigue in quiescent inflammatory bowel disease is linked to *Faecalibacterium prausnitzii* abundance. Gastro Hep Adv. (2025) 4:12. doi: 10.1016/j.gastha.2024.08.012, PMID: 39790237 PMC11713491

[63] Donnino M. Gastrointestinal beriberi: a previously unrecognized syndrome. Ann Intern Med. (2004) 141:898–9. doi: 10.7326/0003-4819-141-11-200412070-00035 15583247

[64] Shah IA Asimi RP Kawoos Y Wani M Saleem T Baba WN. Nonalcoholic Wernicke’s encephalopathy: A retrospective study from a tertiary Care Center in Northern India. J Neurosci Rural Pract. (2017) 8:401–6. doi: 10.4103/jnrp.jnrp_14_17, PMID: 28694620 PMC5488561

[65] Duca J Lum CJ Lo AM. Elevated lactate secondary to gastrointestinal beriberi. J Gen Intern Med. (2015) 31:133–6. doi: 10.1007/s11606-015-3326-2, PMID: 25876741 PMC4699997

[66] Vu TND Junker MS Kurjatko A Albright RC Heller SF Rivera M. Gastrointestinal beriberi mimicking a surgical emergency in a well-nourished patient: A case report. Mayo Clin Proc Innov Qual Outcomes. (2019) 3:506–9. doi: 10.1016/j.mayocpiqo.2019.08.005, PMID: 31993570 PMC6978589

[67] Duca J Lum C. Rare presentation of thiamine deficiency as gastrointestinal syndrome. Hawaii J Med Public Health. (2014) 73:46.

[68] Prakash S. Gastrointestinal beriberi: a forme fruste of Wernicke’s encephalopathy? BMJ Case Rep. (2018) 2018:4841. doi: 10.1136/bcr-2018-224841 PMC6040496 29982183

[69] Williams RD. Observations on induced thiamine (vitamin B1) deficiency in man. Arch Intern Med. (1940) 66:785. doi: 10.1001/archinte.1940.00190160002001

[70] Shimazono N Katsura E. Review of Japanese literature on beriberi and thiamine. Japan: Vitamin B Research Committee of Japan (1965).

[71] Bal B Finelli F Shope T Koch T. Malnutrition-induced dysphagia after roux en Y gastric bypass surgery: 440. Off J Am Coll Gastroenterol ACG. (2010) 105:S161. doi: 10.14309/00000434-201010001-00440, PMID: 39449940

[72] Machado J Ministro P Cancela E Araújo R Castanheira A Silva A. Acute neurologic disorder in Crohn’s disease: A rare life-threatening complication. GE J Port Gastrenterol. (2014) 21:31–4. doi: 10.1016/j.jpg.2013.10.006, PMID: 40111039

[73] Truedsson M Ohlsson B Sjöberg K. Wernicke’s encephalopathy presenting with severe dysphagia: a case report. Alcohol Alcohol. (2002) 37:295–6. doi: 10.1093/alcalc/37.3.295, PMID: 12003921

[74] Cornea A Lata I Simu M Rosca EC. Wernicke encephalopathy presenting with dysphagia: A case report and systematic literature review. Nutrients. (2022) 14:5294. doi: 10.3390/nu14245294, PMID: 36558453 PMC9788281

[75] Sparks MI Collins EN. The role of vitamin B1 in tonus of the large intestine. Am J Dig Dis. (1935) 2:618–20. doi: 10.1007/BF03000956, PMID: 40110477

[76] Hirose Y Endoh H Maruyama M Ohtake M Aizawa O Miida T. False acute abdomen with lactic acidosis in vitamin B1 deficiency. Nihon Kyukyu Igakukai Zasshi. (1993) 4:339–43. doi: 10.3893/jjaam.4.339, PMID: 26573409

[77] Takai S. Habitual constipation as a sign of infantile Preberiberi and the effect of vitamin B. A clinical experiment and study on a Group of Apparently Healthy lnfants nursed with Arakawa=negative human Milk 66th report of the peroxidase reaction. Tohoku J Exp Med. (1935) 27:589–98. doi: 10.1620/tjem.27.589, PMID: 29467341

[78] Ruiz M. Dilatation of the digestive organs due to vitamin B1 deficiency. Rev Clin Esp. (1954) 53:182–8. PMID: 13195060

[79] Du W Lu L Liu Y Yan Y La R Wu Q. The association between dietary vitamin B1 intake and constipation: a population-based study. BMC Gastroenterol. (2024) 24:171. doi: 10.1186/s12876-024-03255-2, PMID: 38760704 PMC11100033

[80] Levin LG Mal’tsev GI Gapparov MM. Effect of thiamine deficiency in hydrochloric acid secretion in the stomach. Vopr Pitan. (1978) 1:36–40. PMID: 716330

[81] Konstam G Sinclair HM. Cardiovascular disturbances caused by deficiency of vitamin B1. Br Heart J. (1940) 2:231–40. doi: 10.1136/hrt.2.4.231, PMID: 18609854 PMC503432

[82] Wilbur DL. The effects of vitamin deficiency on the gastro-intestinal tract. Am J Dig Dis. (1939) 6:610–7. doi: 10.1007/BF02996333

[83] Haden RL. Clinical deficiency disease. Trans Am Clin Climatol Assoc. (1935) 51:238–46. PMID: 21407511 PMC2242086

[84] Hawk EA Hundley JM. Effect of certain B vitamin deficiencies on gastric secretion in the rat. Proc Soc Exp Biol Med. (1951) 78:318–22. doi: 10.3181/00379727-78-19060, PMID: 14892006

[85] Dalldorf G Kellogg M. Incidence of gastric ulcer in albino rats fed diets deficient in vitamin B1. J Exp Med. (1932) 56:391–8. doi: 10.1084/jem.56.3.391, PMID: 19870073 PMC2132102

[86] Iwai W Abe Y Iijima K Koike T Uno K Asano N. Gastric hypochlorhydria is associated with an exacerbation of dyspeptic symptoms in female patients. J Gastroenterol. (2013) 48:214–21. doi: 10.1007/s00535-012-0634-8, PMID: 22829345

[87] Zhang X Chen J Fu K Huang X. Clinical observation on the treatment of functional dyspepsia by injection of vitamin B1 into Zusanli and Hegu acupoints. Acupunct Res. (2021) 46:1043–7. doi: 10.13702/j.1000-0607.20210660, PMID: 34970882

[88] Ianiro G Pecere S Giorgio V Gasbarrini A Cammarota G. Digestive enzyme supplementation in gastrointestinal diseases. Curr Drug Metab. (2016) 17:187–93. doi: 10.2174/138920021702160114150137, PMID: 26806042 PMC4923703

[89] Prasannan KG Sundaresan R Venkatesan D. Thiamine deficency and protein secretion by pancreatic slices in vitro. Experientia. (1977) 33:169–70. doi: 10.1007/BF02124046, PMID: 844540

[90] Srinivasan P Thrower EC Loganathan G Balamurugan AN Subramanian VS Gorelick FS. Chronic nicotine exposure in vivo and in vitro inhibits vitamin B1 (thiamin) uptake by pancreatic acinar cells. PLoS One. (2015) 10:e0143575. doi: 10.1371/journal.pone.0143575, PMID: 26633299 PMC4669105

[91] Subramanya SB Subramanian VS Sekar VT Said HM. Thiamin uptake by pancreatic acinar cells: effect of chronic alcohol feeding/exposure. Am J Physiol Gastrointest Liver Physiol. (2011) 301:G896–904. doi: 10.1152/ajpgi.00308.2011, PMID: 21868632 PMC3220324

[92] Menendez G Vazquez E. Carencia de B en patologia digestiva [B deficiency in digestive pathology]. Rev Clín Esp. (1944) 14:241–6.

[93] Plana GA. Effect of vitamin B1 on the enzymes of the pancreas in children. Riv Clin Pediatr. (1939) 1:353–9.

[94] Singh M. Effect of thiamin deficiency on pancreatic acinar cell function. Am J Clin Nutr. (1982) 36:500–4. doi: 10.1093/ajcn/36.3.500, PMID: 6180623

[95] Khoso MS Saeed A Fayyaz Z Goraya A Anjum MN Cheema HA. Severe acute pancreatitis complicating with pancreatic encephalopathy in an adolescent girl. Pak Pediatr J. (2023) 47:11.

[96] Chen L Shu Y Liang X Chen EC Yee SW Zur AA. OCT1 is a high-capacity thiamine transporter that regulates hepatic steatosis and is a target of metformin. Proc Natl Acad Sci. (2014) 111:9983–8. doi: 10.1073/pnas.1314939111, PMID: 24961373 PMC4103324

[97] Laforenza U Patrini C Alvisi C Faelli A Licandro A Rindi G. Thiamine uptake in human intestinal biopsy specimens, including observations from a patient with acute thiamine deficiency. Am J Clin Nutr. (1997) 66:320–6. doi: 10.1093/ajcn/66.2.320, PMID: 9250110

[98] Zhang K Huentelman MJ Rao F Sun EI Corneveaux JJ Schork AJ. Genetic implication of a novel thiamine transporter in human hypertension. J Am Coll Cardiol. (2014) 63:1542–55. doi: 10.1016/j.jacc.2014.01.007, PMID: 24509276 PMC3992204

[99] Kato K Mori H Kito T Yokochi M Ito S Inoue K. Investigation of endogenous compounds for assessing the drug interactions in the urinary excretion involving multidrug and toxin extrusion proteins. Pharm Res. (2014) 31:136–47. doi: 10.1007/s11095-013-1144-y 23907530

[100] Rindi G Laforenza U. Thiamine intestinal transport and related issues: recent aspects. Proc Soc Exp Biol Med. (2000) 224:246–55. doi: 10.1046/j.1525-1373.2000.22428.x, PMID: 10964259

[101] Rindi G Ricci V Gastaldi G Patrini C. Intestinal alkaline phosphatase can Transphosphorylate thiamin to thiamin monophosphate during intestinal transport in the rat. Arch Physiol Biochem. (1995) 103:33–8. doi: 10.3109/13813459509007560, PMID: 8574774

[102] Tanihara Y Masuda S Sato T Katsura T Ogawa O Inui K-I. Substrate specificity of MATE1 and MATE2-K, human multidrug and toxin extrusions/H(+)-organic cation antiporters. Biochem Pharmacol. (2007) 74:359–71. doi: 10.1016/j.bcp.2007.04.010, PMID: 17509534

[103] Laforenza U Gastaldi G Rindi G. Thiamine outflow from the enterocyte: a study using basolateral membrane vesicles from rat small intestine. J Physiol. (1993) 468:401–12. doi: 10.1113/jphysiol.1993.sp019778, PMID: 8254515 PMC1143833

[104] Laforenza U Orsenigo MN Rindi G. A thiamine/H+ Antiport mechanism for thiamine entry into brush border membrane vesicles from rat small intestine. J Membr Biol. (1998) 161:151–61. doi: 10.1007/s002329900322, PMID: 9435271

[105] Otsuka M Matsumoto T Morimoto R Arioka S Omote H Moriyama Y. A human transporter protein that mediates the final excretion step for toxic organic cations. Proc Natl Acad Sci. (2005) 102:17923–8. doi: 10.1073/pnas.0506483102, PMID: 16330770 PMC1312386

[106] Komatsu T Hiasa M Miyaji T Kanamoto T Matsumoto T Otsuka M. Characterization of the human MATE2 proton-coupled polyspecific organic cation exporter. Int J Biochem Cell Biol. (2011) 43:913–8. doi: 10.1016/j.biocel.2011.03.005, PMID: 21419862

[107] Said HM Balamurugan K Subramanian VS Marchant JS. Expression and functional contribution of hTHTR-2 in thiamin absorption in human intestine. Am J Physiol Gastrointest Liver Physiol. (2004) 286:G491–8. doi: 10.1152/ajpgi.00361.2003, PMID: 14615284

[108] Subramanian VS Marchant JS Said HM. Targeting and trafficking of the human thiamine transporter-2 in epithelial cells. J Biol Chem. (2006) 281:5233–45. doi: 10.1074/jbc.M512765200, PMID: 16371350

[109] Gabriel F Spriestersbach L Fuhrmann A Jungnickel KEJ Mostafavi S Pardon E. Structural basis of thiamine transport and drug recognition by SLC19A3. Nat Commun. (2024) 15:8542. doi: 10.1038/s41467-024-52872-8, PMID: 39358356 PMC11447181

[110] Gupta A Thelma BK. Identification of critical variants within SLC44A4, an ulcerative colitis susceptibility gene identified in a GWAS in north Indians. Genes Immun. (2016) 17:105–9. doi: 10.1038/gene.2015.53, PMID: 26741288

[111] Gupta A Juyal G Sood A Midha V Yamazaki K Vich Vila A. A cross-ethnic survey of CFB and SLC44A4, Indian ulcerative colitis GWAS hits, underscores their potential role in disease susceptibility. Eur J Hum Genet. (2016) 25:111–22. doi: 10.1038/ejhg.2016.131, PMID: 27759029 PMC5159766

[112] Wu J Cheng Y Zhang R Shen H Ma L Yang J. Evaluating the Association of Common Variants of the SLC44A4 gene with ulcerative colitis susceptibility in the Han Chinese population. Genet Test Mol Biomark. (2017) 21:555–9. doi: 10.1089/gtmb.2017.0010, PMID: 28753073

[113] Juyal G Negi S Sood A Gupta A Prasad P Senapati S. Genome-wide association scan in north Indians reveals three novel HLA-independent risk loci for ulcerative colitis. Gut. (2015) 64:571–9. doi: 10.1136/gutjnl-2013-306625, PMID: 24837172

[114] Christensen J El-Gebali S Natoli M Sengstag T Delorenzi M Bentz S. Defining new criteria for selection of cell-based intestinal models using publicly available databases. BMC Genomics. (2012) 13:274. doi: 10.1186/1471-2164-13-274, PMID: 22726358 PMC3412164

[115] Vomhof-DeKrey EE Singhal S Singhal SK Stover AD Rajpathy O Preszler E. RNA sequencing of intestinal enterocytes pre- and post-roux-en-Y gastric bypass reveals alteration in gene expression related to enterocyte differentiation, restitution, and obesity with regulation by Schlafen 12. Cells. (2022) 11:3283. doi: 10.3390/cells11203283, PMID: 36291149 PMC9601224

[116] Bjerrum JT Hansen M Olsen J Nielsen OH. Genome-wide gene expression analysis of mucosal colonic biopsies and isolated colonocytes suggests a continuous inflammatory state in the lamina propria of patients with quiescent ulcerative colitis. Inflamm Bowel Dis. (2010) 16:999–1007. doi: 10.1002/ibd.21142 19834973

[117] Nabokina SM Inoue K Subramanian VS Valle JE Yuasa H Said HM. Molecular identification and functional characterization of the human colonic thiamine pyrophosphate transporter. J Biol Chem. (2014) 289:4405–16. doi: 10.1074/jbc.M113.528257, PMID: 24379411 PMC3924303

[118] Dang Y Zhou D Du X Zhao H Lee C-H Yang J. Molecular mechanism of substrate recognition by folate transporter SLC19A1. Cell Discov. (2022) 8:141–11. doi: 10.1038/s41421-022-00508-w, PMID: 36575193 PMC9794768

[119] Zhao R Goldman ID. Folate and thiamine transporters mediated by facilitative carriers (SLC19A1-3 and SLC46A1) and folate receptors. Mol Asp Med. (2013) 34:373–85. doi: 10.1016/j.mam.2012.07.006, PMID: 23506878 PMC3831518

[120] Cohen E Margalit I Shochat T Goldberg E Krause I. Sex differences in folate levels: A cross sectional study of a large cohort from Israel. Isr Med Assoc J. (2021) 23:17–22. PMID: 33443337

[121] Hao L Ma J Stampfer MJ Ren A Tian Y Tang Y. Geographical, seasonal and gender differences in folate status among Chinese adults. J Nutr. (2003) 133:3630–5. doi: 10.1093/jn/133.11.3630, PMID: 14608086

[122] Kim Y-N Cho Y-O. Folate food source, usual intake, and folate status in Korean adults. Nutr Res Pract. (2018) 12:47–51. doi: 10.4162/nrp.2018.12.1.47, PMID: 29399296 PMC5792256

[123] Wang X Wang Y Ma X Zhou S Xu J Guo Y. Gender-specific association of SLC19A1 and MTHFR genetic polymorphism with oxidative stress biomarkers and plasma folate levels in older adults. Exp Gerontol. (2023) 178:112208. doi: 10.1016/j.exger.2023.112208, PMID: 37201763

[124] Mataix J Aranda P Sánchez C Montellano MA Planells E Llopis J. Assessment of thiamin (vitamin B1) and riboflavin (vitamin B2) status in an adult Mediterranean population. Br J Nutr. (2003) 90:661–6. doi: 10.1079/BJN2003926, PMID: 13129473

[125] O’Brien NL Quadri G Lightley I Sharp SI Guerrini I Smith I. SLC19A1 genetic variation leads to altered thiamine diphosphate transport: implications for the risk of developing Wernicke–Korsakoff’s syndrome. Alcohol Alcohol. (2022) 57:581–8. doi: 10.1093/alcalc/agac032, PMID: 35952336

[126] Abdul-Muneer PM Alikunju S Schuetz H Szlachetka AM Ma X Haorah J. Impairment of thiamine transport at the GUT-BBB-AXIS contributes to Wernicke’s encephalopathy. Mol Neurobiol. (2018) 55:5937–50. doi: 10.1007/s12035-017-0811-0, PMID: 29128903 PMC9420083

[127] Greenwood J Love ER Pratt OE. Kinetics of thiamine transport across the blood-brain barrier in the rat. J Physiol. (1982) 327:95–103. doi: 10.1113/jphysiol.1982.sp014222, PMID: 7120152 PMC1225099

[128] Bettendorff L Wins P. Thiamine triphosphatase and the CYTH superfamily of proteins. FEBS J. (2013) 280:6443–55. doi: 10.1111/febs.12498, PMID: 24021036

[129] Bettendorff L. Update on thiamine Triphosphorylated derivatives and metabolizing enzymatic complexes. Biomol Ther. (2021) 11:1645. doi: 10.3390/biom11111645, PMID: 34827643 PMC8615392

[130] Hurt JK Coleman JL Fitzpatrick BJ Taylor-Blake B Bridges AS Vihko P. Prostatic acid phosphatase is required for the Antinociceptive effects of thiamine and Benfotiamine. PLoS One. (2012) 7:e48562. doi: 10.1371/journal.pone.0048562, PMID: 23119057 PMC3485352

[131] Zylka MJ Sowa NA Taylor-Blake B Twomey MA Herrala A Voikar V. Prostatic acid phosphatase is an ectonucleotidase and suppresses pain by generating adenosine. Neuron. (2008) 60:111–22. doi: 10.1016/j.neuron.2008.08.024, PMID: 18940592 PMC2629077

[132] Knyihár-Csillik E Bezzegh A Böti S Csillik B. Thiamine monophosphatase: a genuine marker for transganglionic regulation of primary sensory neurons. J Histochem Cytochem. (1986) 34:363–71. doi: 10.1177/34.3.3005391, PMID: 3005391

[133] Kim YJ Lee Y Kasimoglu Y Seymen F Simmer JP Hu JC-C. Recessive mutations in ACP4 cause Amelogenesis Imperfecta. J Dent Res. (2022) 101:37–45. doi: 10.1177/00220345211015119, PMID: 34036831 PMC8721729

[134] Yousef GM Diamandis M Jung K Diamandis EP. Molecular cloning of a novel human acid phosphatase gene (ACPT) that is highly expressed in the testis. Genomics. (2001) 74:385–95. doi: 10.1006/geno.2001.6556, PMID: 11414767

[135] Hiroyama M Takenawa T. Isolation of a cDNA encoding human lysophosphatidic acid phosphatase that is involved in the regulation of mitochondrial lipid biosynthesis. J Biol Chem. (1999) 274:29172–80. doi: 10.1074/jbc.274.41.29172, PMID: 10506173

[136] Nadler HL Egan TJ. Deficiency of lysosomal acid phosphatase. N Engl J Med. (1970) 282:302–7. doi: 10.1056/NEJM197002052820604, PMID: 5410815

[137] Whitfield KC Bourassa MW Adamolekun B Bergeron G Bettendorff L Brown KH. Thiamine deficiency disorders: diagnosis, prevalence, and a roadmap for global control programs. Ann N Y Acad Sci. (2018) 1430:3–43. doi: 10.1111/nyas.13919, PMID: 30151974 PMC6392124

[138] Lausch E Janecke A Bros M Trojandt S Alanay Y De Laet C. Genetic deficiency of tartrate-resistant acid phosphatase associated with skeletal dysplasia, cerebral calcifications and autoimmunity. Nat Genet. (2011) 43:132–7. doi: 10.1038/ng.749, PMID: 21217752

[139] Marchetti V Menghini R Rizza S Vivanti A Feccia T Lauro D. Benfotiamine counteracts glucose toxicity effects on endothelial progenitor cell differentiation via Akt/FoxO signaling. Diabetes. (2006) 55:2231–7. doi: 10.2337/db06-0369, PMID: 16873685

[140] Aleshin VA Mezhenska OA Parkhomenko YM Kaehne T Bunik VI. Thiamine mono- and diphosphate phosphatases in bovine brain Synaptosomes. Biochem Mosc. (2020) 85:378–86. doi: 10.1134/S000629792003013X, PMID: 32564742

[141] Bunik VI Aleshin VA. Chapter 11 - analysis of the protein binding sites for thiamin and its derivatives to elucidate the molecular mechanisms of the noncoenzyme action of thiamin (vitamin B1) In: Rahman A, editor. Studies in natural products chemistry. Amsterdam, Netherlands: Elsevier (2017). 375–429.

[142] Aleshin VA Mkrtchyan GV Bunik VI. Mechanisms of non-coenzyme action of thiamine: protein targets and medical significance. Biochem Mosc. (2019) 84:829–50. doi: 10.1134/S0006297919080017, PMID: 31522667

[143] Parkhomenko YM Pavlova AS Mezhenskaya OA. Mechanisms responsible for the high sensitivity of neural cells to vitamin B1 deficiency. Neurophysiology. (2016) 48:429–48. doi: 10.1007/s11062-017-9620-3

[144] Gangolf M Czerniecki J Radermecker M Detry O Nisolle M Jouan C. Thiamine status in humans and content of phosphorylated thiamine derivatives in biopsies and cultured cells. PLoS One. (2010) 5:e13616. doi: 10.1371/journal.pone.0013616, PMID: 21049048 PMC2963613

[145] Gutiérrez-Rey M Castellar-Visbal L Acevedo-Vergara K Vargas-Manotas J Rivera-Porras D Londoño-Juliao G. The weight of bariatric surgery: Wernicke-Korsakoff syndrome after vertical sleeve gastrectomy-A case series. J Pers Med. (2024) 14:638. doi: 10.3390/jpm14060638, PMID: 38929859 PMC11204981

[146] Parrott JM Parrott AJ Parrott JS Williams NN Dumon KR. Predicting recurrent deficiency and suboptimal monitoring of thiamin deficiency in patients with metabolic and bariatric surgery. Nutrients. (2024) 16:2226. doi: 10.3390/nu16142226, PMID: 39064668 PMC11280029

[147] Kairis S Andris C Rakic J-M. Wernicke encephalopathy: a neuro-ophthalmological side effect of bariatric surgery. Rev Med Liege. (2024) 79:492–6. PMID: 39129546

[148] Ben-Zvi D Meoli L Abidi WM Nestoridi E Panciotti C Castillo E. Time-dependent molecular responses differ between gastric bypass and dieting but are conserved across species. Cell Metab. (2018) 28:310–323.e6. doi: 10.1016/j.cmet.2018.06.004, PMID: 30043755 PMC6628900

[149] Mkrtchyan G Graf A Bettendorff L Bunik V. Cellular thiamine status is coupled to function of mitochondrial 2-oxoglutarate dehydrogenase. Neurochem Int. (2016) 101:66–75. doi: 10.1016/j.neuint.2016.10.009, PMID: 27773789

[150] Aleshin VA Artiukhov AV Oppermann H Kazantsev AV Lukashev NV Bunik VI. Mitochondrial impairment may increase cellular NAD(P)H: Resazurin oxidoreductase activity, perturbing the NAD(P)H-based viability assays. Cells. (2015) 4:427–51. doi: 10.3390/cells4030427, PMID: 26308058 PMC4588044

[151] O’Connell MJ Walworth NC Carr AM. The G2-phase DNA-damage checkpoint. Trends Cell Biol. (2000) 10:296–303. doi: 10.1016/s0962-8924(00)01773-6 10856933

[152] Nabokina SM Reidling JC Said HM. Differentiation-dependent up-regulation of intestinal thiamin uptake: cellular and molecular mechanisms. J Biol Chem. (2005) 280:32676–82. doi: 10.1074/jbc.M505243200, PMID: 16055442

[153] Weidinger A Milivojev N Hosmann A Duvigneau JC Szabo C Törö G. Oxoglutarate dehydrogenase complex controls glutamate-mediated neuronal death. Redox Biol. (2023) 62:102669. doi: 10.1016/j.redox.2023.102669, PMID: 36933393 PMC10031542

[154] Boyko A Tsepkova P Aleshin V Artiukhov A Mkrtchyan G Ksenofontov A. Severe spinal cord injury in rats induces chronic changes in the spinal cord and cerebral cortex metabolism, adjusted by thiamine that improves locomotor performance. Front Mol Neurosci. (2021) 14:593. doi: 10.3389/fnmol.2021.620593, PMID: 33867932 PMC8044794

[155] Artiukhov AV Solovjeva ON Balashova NV Sidorova OP Graf AV Bunik VI. Pharmacological doses of thiamine benefit patients with the Charcot–Marie–tooth neuropathy by changing thiamine diphosphate levels and affecting regulation of thiamine-dependent enzymes. Biochem Mosc. (2024) 89:1161–82. doi: 10.1134/S0006297924070010, PMID: 39218016

[156] Parkhomenko YM Kudryavtsev PA Pylypchuk SY Chekhivska LI Stepanenko SP Sergiichuk AA. Chronic alcoholism in rats induces a compensatory response, preserving brain thiamine diphosphate, but the brain 2-oxo acid dehydrogenases are inactivated despite unchanged coenzyme levels. J Neurochem. (2011) 117:1055–65. doi: 10.1111/j.1471-4159.2011.07283.x, PMID: 21517848

[157] Nixon PF Diefenbach RJ Duggleby RG. Inhibition of transketolase and pyruvate decarboxylase by omeprazole. Biochem Pharmacol. (1992) 44:177–9. doi: 10.1016/0006-2952(92)90053-L, PMID: 1632833

[158] Nemeria NS Shome B DeColli AA Heflin K Begley TP Meyers CF. Competence of thiamin diphosphate-dependent enzymes with 2′-Methoxythiamin diphosphate derived from Bacimethrin, a naturally occurring thiamin anti-vitamin. Biochemistry. (2016) 55:1135–48. doi: 10.1021/acs.biochem.5b01300, PMID: 26813608 PMC4852132

[159] Iwadate D Sato K Kanzaki M Komiyama C Watanabe C Eguchi T. Thiamine deficiency in metronidazole-induced encephalopathy: A metabolic correlation? J Neurol Sci. (2017) 379:324–6. doi: 10.1016/j.jns.2017.06.042 28716273

[160] Alston TA Abeles RH. Enzymatic conversion of the antibiotic metronidazole to an analog of thiamine. Arch Biochem Biophys. (1987) 257:357–62. doi: 10.1016/0003-9861(87)90577-7, PMID: 2821910

[161] Agyei-Owusu K Leeper FJ. Thiamin diphosphate in biological chemistry: analogues of thiamin diphosphate in studies of enzymes and riboswitches. FEBS J. (2009) 276:2905–16. doi: 10.1111/j.1742-4658.2009.07018.x, PMID: 19490097

[162] Sheikh A Tumala B Vickers TJ Martin JC Rosa BA Sabui S. Enterotoxigenic *Escherichia coli* heat-labile toxin drives enteropathic changes in small intestinal epithelia. Nat Commun. (2022) 13:6886. doi: 10.1038/s41467-022-34687-7, PMID: 36371425 PMC9653437

[163] Anandam KY Srinivasan P Yasujima T Al-Juburi S Said HM. Proinflammatory cytokines inhibit thiamin uptake by human and mouse pancreatic acinar cells: involvement of transcriptional mechanism(s). Am J Physiol Gastrointest Liver Physiol. (2021) 320:G108–16. doi: 10.1152/ajpgi.00361.2020, PMID: 33146542 PMC8112188

[164] Anthonymuthu S Sabui S Manzon KI Sheikh A Fleckenstein JM Said HM. Bacterial lipopolysaccharide inhibits free thiamin uptake along the intestinal tract via interference with membrane expression of thiamin transporters 1 and 2. Am J Physiol Cell Physiol. (2024) 327:C1163–77. doi: 10.1152/ajpcell.00570.2024, PMID: 39246143 PMC11559647

[165] Von Muralt A. The role of thiamine (vitamin B1) in nervous excitation. Exp Cell Res. (1958) 14:72–9. PMID: 13586284

[166] Von Muralt A. Thiamine and peripheral neurophysiology In: Harris RS Thimann KV, editors. Vitamins and Hormones. Cambridge, MA: Academic Press (1947). 93–118.

[167] Minz B. Sur la liberation de la vitamine B1 par le trone isole de nerf pneumogastrique soumis a l’exitation electrique. CRSoc Biol. (1938) 127:1251–3.

[168] Wessler I Kirkpatrick CJ. Acetylcholine beyond neurons: the non-neuronal cholinergic system in humans. Br J Pharmacol. (2008) 154:1558–71. doi: 10.1038/bjp.2008.185, PMID: 18500366 PMC2518461

[169] Caicedo A. Paracrine and autocrine interactions in the human islet: more than meets the eye. Semin Cell Dev Biol. (2013) 24:11–21. doi: 10.1016/j.semcdb.2012.09.007, PMID: 23022232 PMC3570628

[170] Ghoshal UC Shukla R Ghoshal U. Small intestinal bacterial overgrowth and irritable bowel syndrome: A bridge between functional organic dichotomy. Gut Liver. (2017) 11:196–208. doi: 10.5009/gnl16126, PMID: 28274108 PMC5347643

[171] Husebye E. Gastrointestinal motility disorders and bacterial overgrowth. J Intern Med. (1995) 237:419–27. doi: 10.1111/j.1365-2796.1995.tb01196.x 7714466

[172] Pimentel M Soffer EE Chow EJ Kong Y Lin HC. Lower frequency of MMC is found in IBS subjects with abnormal lactulose breath test, suggesting bacterial overgrowth. Dig Dis Sci. (2002) 47:2639–43. doi: 10.1023/a:1021039032413 12498278

[173] Vantrappen G Janssens J Hellemans J Ghoos Y. The interdigestive motor complex of normal subjects and patients with bacterial overgrowth of the small intestine. J Clin Invest. (1977) 59:1158–66. doi: 10.1172/JCI108740, PMID: 864008 PMC372329

[174] Kashyap P Farrugia G. Enteric autoantibodies and gut motility disorders. Gastroenterol Clin N Am. (2008) 37:397–410. doi: 10.1016/j.gtc.2008.02.005, PMID: 18499027 PMC2448392

[175] Therrien A Bouchard S Sidani S Bouin M. Prevalence of small intestinal bacterial overgrowth among chronic pancreatitis patients: A case-control study. Can J Gastroenterol Hepatol. (2016) 2016:7424831–7. doi: 10.1155/2016/7424831, PMID: 27446865 PMC4904664

[176] Liu S Miriyala S Keaton MA Jordan CT Wiedl C Clair DKS. Metabolic effects of acute thiamine depletion are reversed by rapamycin in breast and leukemia cells. PLoS One. (2014) 9:e85702. doi: 10.1371/journal.pone.0085702, PMID: 24454921 PMC3893258

[177] Waldenlind L. Possible role of thiamine in neuromuscular transmission. Acta Physiol Scand. (1979) 105:1–10. doi: 10.1111/j.1748-1716.1979.tb06309.x, PMID: 217239

[178] Vanuytsel T Tack J Farre R. The role of intestinal permeability in gastrointestinal disorders and current methods of evaluation. Front Nutr. (2021) 8:717925. doi: 10.3389/fnut.2021.717925, PMID: 34513903 PMC8427160

[179] Beauchesne E Desjardins P Butterworth RF Hazell AS. Up-regulation of caveolin-1 and blood-brain barrier breakdown are attenuated by N-acetylcysteine in thiamine deficiency. Neurochem Int. (2010) 57:830–7. doi: 10.1016/j.neuint.2010.08.022, PMID: 20816907

[180] Beauchesne É Desjardins P Hazell AS Butterworth RF. Altered expression of tight junction proteins and matrix metalloproteinases in thiamine-deficient mouse brain. Neurochem Int. (2009) 55:275–81. doi: 10.1016/j.neuint.2009.03.014 19576514

[181] Wen L-M Jiang W-D Liu Y Wu P Zhao J Jiang J. Evaluation the effect of thiamin deficiency on intestinal immunity of young grass carp (*Ctenopharyngodon idella*). Fish Shellfish Immunol. (2015) 46:501–15. doi: 10.1016/j.fsi.2015.07.001 26159094

[182] Kunisawa J Sugiura Y Wake T Nagatake T Suzuki H Nagasawa R. Mode of bioenergetic metabolism during B cell differentiation in the intestine determines the distinct requirement for vitamin B1. Cell Rep. (2015) 13:122–31. doi: 10.1016/j.celrep.2015.08.063 26411688

[183] Mantis NJ Rol N Corthésy B. Secretory IgA’s complex roles in immunity and mucosal homeostasis in the gut. Mucosal Immunol. (2011) 4:603–11. doi: 10.1038/mi.2011.41, PMID: 21975936 PMC3774538

[184] Oudman E Wijnia JW Oey MJ van Dam M Postma A. Wernicke’s encephalopathy in Crohn’s disease and ulcerative colitis. Nutrition. (2021) 86:111182. doi: 10.1016/j.nut.2021.111182, PMID: 33611107

[185] de Sire R Rispo A Compare D Tortora F Nardone G Castiglione F. Wernicke encephalopathy in ulcerative colitis. Inflamm Bowel Dis. (2022) 28:e70–1. doi: 10.1093/ibd/izab296 35512144

[186] Yoon SM. Micronutrient deficiencies in inflammatory bowel disease: trivial or crucial? Intest Res. (2016) 14:109–10. doi: 10.5217/ir.2016.14.2.109, PMID: 27175110 PMC4863043

[187] Mańkowska-Wierzbicka D Michalak S Karczewski J Dobrowolska A Wierzbicka A Stelmach-Mardas M. Erythrocyte transketolase deficiency in patients suffering from Crohn’s disease. Eur Rev Med Pharmacol Sci. (2019) 23:8501–5. doi: 10.26355/eurrev_201910_19163 31646581

[188] Pan X Ren Z Liang W Dong X Li J Wang L. Thiamine deficiency aggravates experimental colitis in mice by promoting glycolytic reprogramming in macrophages. Br J Pharmacol. (2025). doi: 10.1111/bph.17435, PMID: 39890689

[189] Van Welden S Selfridge AC Hindryckx P. Intestinal hypoxia and hypoxia-induced signalling as therapeutic targets for IBD. Nat Rev Gastroenterol Hepatol. (2017) 14:596–611. doi: 10.1038/nrgastro.2017.101, PMID: 28853446

[190] Sabui S Ramamoorthy K Romero JM Simoes RD Fleckenstein JM Said HM. Hypoxia inhibits colonic uptake of the microbiota-generated forms of vitamin B1 via HIF-1α-mediated transcriptional regulation of their transporters. J Biol Chem. (2022) 298:562. doi: 10.1016/j.jbc.2022.101562, PMID: 34998824 PMC8800108

[191] Martínez-Moya P Ortega-González M González R Anzola A Ocón B Hernández-Chirlaque C. Exogenous alkaline phosphatase treatment complements endogenous enzyme protection in colonic inflammation and reduces bacterial translocation in rats. Pharmacol Res. (2012) 66:144–53. doi: 10.1016/j.phrs.2012.04.006, PMID: 22569414

[192] Goldberg RF William G Austen J Zhang X Munene G Mostafa G. Intestinal alkaline phosphatase is a gut mucosal defense factor maintained by enteral nutrition. Proc Natl Acad Sci USA. (2008) 105:3551–6. doi: 10.1073/pnas.0712140105, PMID: 18292227 PMC2265168

[193] Gagliani N Palm NW De ZMR Flavell RA. Inflammasomes and intestinal homeostasis: regulating and connecting infection, inflammation and the microbiota. Int Immunol. (2014) 26:495–9. doi: 10.1093/intimm/dxu066, PMID: 24948595 PMC4200027

[194] Achamrah N Déchelotte P Coëffier M. Glutamine and the regulation of intestinal permeability: from bench to bedside. Curr Opin Clin Nutr Metab Care. (2016) 20:86–91. doi: 10.1097/MCO.0000000000000339, PMID: 27749689

[195] Mahmood S Dani HM Mahmood A. Effect of dietary thiamin deficiency on intestinal functions in rats. Am J Clin Nutr. (1984) 40:226–34. doi: 10.1093/ajcn/40.2.226, PMID: 6465054

[196] Araújo WL Trofimova L Mkrtchyan G Steinhauser D Krall L Graf A. On the role of the mitochondrial 2-oxoglutarate dehydrogenase complex in amino acid metabolism. Amino Acids. (2013) 44:683–700. doi: 10.1007/s00726-012-1392-x 22983303

[197] Trofimova LK Araújo WL Strokina AA Fernie AR Bettendorff L Bunik VI. Consequences of the α-ketoglutarate dehydrogenase inhibition for neuronal metabolism and survival: implications for neurodegenerative diseases. Curr Med Chem. (2012) 19:5895–906. doi: 10.2174/092986712804143367, PMID: 23061627

[198] Culliford A Markowitz D Rotterdam H Green PHR. Scalloping of duodenal mucosa in Crohn’s disease. Inflamm Bowel Dis. (2004) 10:270–3. doi: 10.1097/00054725-200405000-00015, PMID: 15290923

[199] Costantini A Pala MI. Thiamine and fatigue in inflammatory bowel diseases: an open-label pilot study. J Altern Complement Med. (2013) 19:704–8. doi: 10.1089/acm.2011.0840, PMID: 23379830

[200] Bager P Hvas CL Rud CL Dahlerup JF. Randomised clinical trial: high-dose oral thiamine versus placebo for chronic fatigue in patients with quiescent inflammatory bowel disease. Aliment Pharmacol Ther. (2021) 53:79–86. doi: 10.1111/apt.16166, PMID: 33210299

[201] Cristina M-O Elizabeth B-R Jose R-AM Berenice P-G Diego Z Luis C-SJ. Mechanisms and therapeutic potential of key anti-inflammatory Metabiotics: trans-Vaccenic acid, Indole-3-lactic acid, thiamine, and butyric acid. Probiotics Antimicrob Proteins. (2025). doi: 10.1007/s12602-025-10475-9, PMID: 39921846

[202] Zhang H Peng AL Zhao FF Yu LH Wang MZ Osorio JS. Thiamine ameliorates inflammation of the ruminal epithelium of Saanen goats suffering from subacute ruminal acidosis. J Dairy Sci. (2020) 103:1931–43. doi: 10.3168/jds.2019-16944, PMID: 31837780

[203] Pan XH Yang L Beckers Y Xue FG Tang ZW Jiang LS. Thiamine supplementation facilitates thiamine transporter expression in the rumen epithelium and attenuates high-grain-induced inflammation in low-yielding dairy cows. J Dairy Sci. (2017) 100:5329–42. doi: 10.3168/jds.2016-11966, PMID: 28501402

[204] Sabui S Anthonymuthu S Ramamoorthy K Skupsky J Jennings TSK Rahmatpanah F. Effect of knocking out mouse Slc44a4 on colonic uptake of the microbiota-generated thiamine pyrophosphate and colon physiology. Am J Physiol Gastrointest Liver Physiol. (2024) 327:G36–46. doi: 10.1152/ajpgi.00065.2024, PMID: 38713615 PMC11376973

[205] Ma Y Elmhadi M Wang C Zhang H Wang H. Dietary supplementation of thiamine down-regulates the expression of mitophagy and endoplasmic reticulum stress-related genes in the rumen epithelium of goats during high-concentrate diet feeding. Ital J Anim Sci. (2021) 20:2220–31. doi: 10.1080/1828051X.2021.1985944, PMID: 40101104

[206] Folda A Scalcon V Tonolo F Rigobello MP Bindoli A. Thiamine disulfide derivatives in thiol redox regulation: role of thioredoxin and glutathione systems. Biofactors. 51:e2121. doi: 10.1002/biof.2121, PMID: 39302148 PMC11681303

[207] Bonaz B Sinniger V Pellissier S. Anti-inflammatory properties of the vagus nerve: potential therapeutic implications of vagus nerve stimulation. J Physiol. (2016) 594:5781–90. doi: 10.1113/JP271539, PMID: 27059884 PMC5063949

[208] Morrisroe K Baron M Frech T Nikpour M. Small intestinal bacterial overgrowth in systemic sclerosis. J Scleroderma Relat Disord. (2020) 5:33–9. doi: 10.1177/2397198319863953, PMID: 35382403 PMC8922590

[209] Kawaguchi Y Nakamura Y Matsumoto I Nishimagi E Satoh T Kuwana M. Muscarinic-3 acetylcholine receptor autoantibody in patients with systemic sclerosis: contribution to severe gastrointestinal tract dysmotility. Ann Rheum Dis. (2009) 68:710–4. doi: 10.1136/ard.2008.096545, PMID: 18762475

[210] Saegusa Y Takeda H Muto S Oridate N Nakagawa K Sadakane C. Decreased motility of the lower esophageal sphincter in a rat model of gastroesophageal reflux disease may be mediated by reductions of serotonin and acetylcholine signaling. Biol Pharm Bull. (2011) 34:704–11. doi: 10.1248/bpb.34.704, PMID: 21532161

[211] Farré R Sifrim D. Regulation of basal tone, relaxation and contraction of the lower oesophageal sphincter. Relevance to drug discovery for oesophageal disorders. Br J Pharmacol. (2007) 153:858–69. doi: 10.1038/sj.bjp.0707572, PMID: 17994108 PMC2267263

[212] Kato R Nakajima K Takahashi T Miyazaki Y Makino T Kurokawa Y. A case of advanced systemic sclerosis with severe GERD successfully treated with acotiamide. Surg Case Rep. (2016) 2:36. doi: 10.1186/s40792-016-0162-5, PMID: 27072944 PMC4829569

[213] Parthasarathy G Ravi K Camilleri M Andrews C Szarka LA Low PA. Effect of neostigmine on gastroduodenal motility in patients with suspected gastrointestinal motility disorders. Neurogastroenterol Motil. (2015) 27:1736–46. doi: 10.1111/nmo.12669, PMID: 26387781 PMC4659742

[214] Jayarajah U Yapa K Ranaweera K Rahuman A Perera P Weerasekara D. Successful use of neostigmine for resistant gastroparesis following distal gastrectomy: A case report. Int J Surg Case Rep. (2023) 106:108166. doi: 10.1016/j.ijscr.2023.108166, PMID: 37068456 PMC10130466

[215] Ollat H Laurent B Bakchine S Michel B-F Touchon J Dubois B. Effets de l’association de la Sulbutiamine à un inhibiteur de l’acétylcholinestérase dans les formes légères à modérées de la maladie d’Alzheimer. L'Encéphale. (2007) 33:211–5. doi: 10.1016/S0013-7006(07)91552-3, PMID: 17675917

[216] Matsueda K Hongo M Tack J Aoki H Saito Y Kato H. Clinical trial: dose-dependent therapeutic efficacy of acotiamide hydrochloride (Z-338) in patients with functional dyspepsia - 100 mg t.i.d. is an optimal dosage. Neurogastroenterol Motil. (2010) 22:618–e173. doi: 10.1111/j.1365-2982.2009.01449.x, PMID: 20059698

[217] Shrestha DB Budhathoki P Subedi P Khadka M Karki P Sedhai YR. Acotiamide and functional dyspepsia: A systematic review and Meta-analysis. Cureus. (2021) 13:e20532. doi: 10.7759/cureus.20532, PMID: 35070565 PMC8765587

[218] Nowlan ML Scott LJ. Acotiamide: first global approval. Drugs. (2013) 73:1377–83. doi: 10.1007/s40265-013-0100-9, PMID: 23881665

[219] Szutowicz A Bielarczyk H Ronowska A Gul-Hinc S Klimaszewska-Łata J Dyś A. Intracellular redistribution of acetyl-CoA, the pivotal point in differential susceptibility of cholinergic neurons and glial cells to neurodegenerative signals. Biochem Soc Trans. (2014) 42:1101–6. doi: 10.1042/BST20140078, PMID: 25110009

[220] Bizon-Zygmańska D Jankowska-Kulawy A Bielarczyk H Pawełczyk T Ronowska A Marszałł M. Acetyl-CoA metabolism in amprolium-evoked thiamine pyrophosphate deficits in cholinergic SN56 neuroblastoma cells. Neurochem Int. (2011) 59:208–16. doi: 10.1016/j.neuint.2011.04.018, PMID: 21672592

[221] Mkrtchyan G Aleshin V Parkhomenko Y Kaehne T Luigi Di Salvo M Parroni A. Molecular mechanisms of the non-coenzyme action of thiamin in brain: biochemical, structural and pathway analysis. Sci Rep. (2015) 5:12583. doi: 10.1038/srep12583, PMID: 26212886 PMC4515825

[222] Szutowicz A Bielarczyk H Jankowska-Kulawy A Pawełczyk T Ronowska A. Acetyl-CoA the key factor for survival or death of cholinergic neurons in course of neurodegenerative diseases. Neurochem Res. (2013) 38:1523–42. doi: 10.1007/s11064-013-1060-x, PMID: 23677775 PMC3691476

[223] Szutowicz A Madziar B Pawełczyk T Tomaszewicz M Bielarczyk H. Effects of NGF on acetylcholine, acetyl-CoA metabolism, and viability of differentiated and non-differentiated cholinergic neuroblastoma cells. J Neurochem. (2004) 90:952–61. doi: 10.1111/j.1471-4159.2004.02556.x, PMID: 15287901

[224] Madziar B Tomaszewicz M Matecki A Bielarczyk H Szutowicz A. Interactions between p75 and TrkA receptors in differentiation and vulnerability of SN56 cholinergic cells to beta-amyloid. Neurochem Res. (2003) 28:461–5. doi: 10.1023/a:1022800802179, PMID: 12675131

[225] Wada-Takahashi S Tamura K. Actions of reactive oxygen species on AH/type 2 myenteric neurons in guinea pig distal colon. Am J Physiol Gastrointest Liver Physiol. (2000) 279:G893–902. doi: 10.1152/ajpgi.2000.279.5.G893, PMID: 11052985

[226] Brown IAM McClain JL Watson RE Patel BA Gulbransen BD. Enteric glia mediate neuron death in colitis through purinergic pathways that require Connexin-43 and nitric oxide. Cell Mol Gastroenterol Hepatol. (2016) 2:77–91. doi: 10.1016/j.jcmgh.2015.08.007, PMID: 26771001 PMC4707972

[227] Karuppagounder SS Xu H Shi Q Chen LH Pedrini S Pechman D. Thiamine deficiency induces oxidative stress and exacerbates the plaque pathology in Alzheimer’s mouse model. Neurobiol Aging. (2009) 30:1587–600. doi: 10.1016/j.neurobiolaging.2007.12.013, PMID: 18406011 PMC2782730

[228] Campanucci VA Krishnaswamy A Cooper E. Mitochondrial reactive oxygen species inactivate neuronal nicotinic acetylcholine receptors and induce Long-term depression of fast nicotinic synaptic transmission. J Neurosci. (2008) 28:1733–44. doi: 10.1523/JNEUROSCI.5130-07.2008, PMID: 18272694 PMC6671543

[229] Stavely R Abalo R Nurgali K. Targeting enteric neurons and Plexitis for the Management of Inflammatory Bowel Disease. Curr Drug Targets. (2020) 21:1428–39. doi: 10.2174/1389450121666200516173242, PMID: 32416686

[230] Lakhan SE Kirchgessner A. Neuroinflammation in inflammatory bowel disease. J Neuroinflammation. (2010) 7:37. doi: 10.1186/1742-2094-7-37, PMID: 20615234 PMC2909178

[231] Sasa M Takemoto I Nishino K Itokawa Y. The role of thiamine on excitable membrane of crayfish Giant axon. J Nutr Sci Vitaminol (Tokyo). (1976) 22 SUPPL:21–4. doi: 10.3177/jnsv.22.Supplement_21, PMID: 978275

[232] Nghiem H-O Bettendorff L Changeux J-P. Specific phosphorylation of Torpedo 43K rapsyn by endogenous kinase(s) with thiamine triphosphate as the phosphate donor. FASEB J. (2000) 14:543–54. doi: 10.1096/fasebj.14.3.543, PMID: 10698970

[233] Parkhomenko IM Strokina AA Pilipchuk SI Stepanenko SP Chekhovskaia LI Donchenko GV. Existence of two different active sites on thiamine binding protein in plasma membranes of synaptosomes. Ukr Biokhimichnyi Zh 1999. (2010) 82:34–41. PMID: 20684226

[234] Parkhomenko YM Vovk AI Protasova ZS Pylypchuk SY Chorny SA Pavlova OS. Thiazolium salt mimics the non-coenzyme effects of vitamin B1 in rat synaptosomes. Neurochem Int. (1999) 178:105791. doi: 10.1016/j.neuint.2024.105791, PMID: 38880231

[235] Yamashita A Shimamoto N Morita K Sugiyama H Kimoto M Toda K. Thiamine and quinine differently inhibit the early phase of acetylcholine-dependent contraction of mouse ileum in vitro. Int J Nutr Food Sci. (2018) 7:94–9. doi: 10.11648/j.ijnfs.20180703.13

[236] Yamashita A Shimamoto N Morita K Sugiyama H Tadakuma S Kato M. Differential inhibition of the rhythm and amplitude of acetylcholine-dependent contraction in the murine jejunum and ileum in vitro by thiamin and quinine. J Food Nutr Sci. (2018) 6:115–22. doi: 10.11648/j.jfns.20180605.11

[237] Waldenlind L Elfman L Rydqvist B. Binding of thiamine to nicotinic acetylcholine receptor in Torpedo marmorata and the frog end plate. Acta Physiol Scand. (1978) 103:154–9. doi: 10.1111/j.1748-1716.1978.tb06202.x, PMID: 307899

[238] Hirsch JA Parrott J. New considerations on the neuromodulatory role of thiamine. Pharmacology. (2012) 89:111–6. doi: 10.1159/000336339, PMID: 22398704

[239] Bai L Mesgarzadeh S Ramesh KS Huey EL Liu Y Gray LA. Genetic identification of vagal sensory neurons that control feeding. Cell. (2019) 179:1129–1143.e23. doi: 10.1016/j.cell.2019.10.031, PMID: 31730854 PMC6916730

[240] Sinclair R Bajekal RR. Vagal nerve stimulation and reflux. Anesth Analg. (2007) 105:884–5. doi: 10.1213/01.ane.0000269687.74660.45 17717266

[241] Lu K-H Cao J Oleson S Ward MP Phillips R Powley TL. Vagus nerve stimulation promotes gastric emptying by increasing pyloric opening measured with magnetic resonance imaging. Neurogastroenterol Motil. (2018) 30:e13380. doi: 10.1111/nmo.13380, PMID: 29797377 PMC6160317

[242] Paulon E Nastou D Jaboli F Marin J Liebler E Epstein O. Proof of concept: short-term non-invasive cervical vagus nerve stimulation in patients with drug-refractory gastroparesis. Frontline Gastroenterol. (2017) 8:325–30. doi: 10.1136/flgastro-2017-100809, PMID: 29067158 PMC5641854

[243] Costantini TW Bansal V Krzyzaniak M Putnam JG Peterson CY Loomis WH. Vagal nerve stimulation protects against burn-induced intestinal injury through activation of enteric glia cells. Am J Physiol. (2010) 299:G1308–18. doi: 10.1152/ajpgi.00156.2010, PMID: 20705905 PMC3774266

[244] Rao M Gershon MD. The bowel and beyond: the enteric nervous system in neurological disorders. Nat Rev Gastroenterol Hepatol. (2016) 13:517–28. doi: 10.1038/nrgastro.2016.107, PMID: 27435372 PMC5005185

[245] Fleming MA II Ehsan L Moore SR Levin DE. The enteric nervous system and its emerging role as a therapeutic target. Gastroenterol Res Pract. (2020) 2020:8024171–13. doi: 10.1155/2020/8024171, PMID: 32963521 PMC7495222

[246] Jakob MO Murugan S Klose CSN. Neuro-immune circuits regulate immune responses in tissues and organ homeostasis. Front Immunol. (2020) 11:308. doi: 10.3389/fimmu.2020.00308, PMID: 32265899 PMC7099652

[247] Romanenko AV Gnatenko VM Vladimirova IA. Effect of thiamine on neuromuscular transmission in smooth muscles. Neurophysiology. (1994) 26:370–7. doi: 10.1007/BF01053581, PMID: 40110477

[248] Fujioka F Choki S Suzuki T Sahashi Y. Stimulating effect of thiamine and its derivatives on rat intestinal muscular contraction. Vitamins. (1966) 34:390–2. doi: 10.20632/vso.34.4_390

[249] Hukuhara T Fukuda H. The effects of thiamine Tetrahydrofurfuryl disulfide upon the movement of the isolated small intestine. J Vitaminol (Kyoto). (1965) 11:253–60. doi: 10.5925/jnsv1954.11.253, PMID: 5883246

[250] Saakyan AG Arutyunova NL Nikolaeva EA Saakyan AG Arutyunova NL Nikolaeva EA. Effect of vitamins B1, B6, B12, and C on the motor activity of the stomach, small and large intestines in patients with chronic enterocolitis and colitis. Vopr Pitan. (1964) 23:45–50. 14255523

[251] Tsuchida S Kimura Y. Electromyography of the intestines by the Intraintestinal method. Tohoku J Exp Med. (1966) 89:61–8. doi: 10.1620/tjem.89.61, PMID: 5914092

[252] Nakayama S Nanba R. Effect of thiamine Tetrahydrofurfuryldisulfide on the peristalsis of the small bowel. Vitamins. (1963) 28:235–7. doi: 10.20632/vso.28.3_235

[253] Hukuhara T Nanba R Siina H. Effect of thiamine Tetrahydrofurfuryl disulfide upon the intestinal motility. J Vitaminol (Kyoto). (1965) 11:210–4. doi: 10.5925/jnsv1954.11.210, PMID: 5859678

[254] Hills JI Golub MS Bettendorff L Keen CL. The effect of thiamin tetrahydrofurfuryl disulfide on behavior of juvenile DBA/2J mice. Neurotoxicol Teratol. (2012) 34:242–52. doi: 10.1016/j.ntt.2011.07.006, PMID: 21816221

[255] Lonsdale D Shamberger RJ Audhya T. Treatment of autism spectrum children with thiamine tetrahydrofurfuryl disulfide: a pilot study. Neuro Endocrinol Lett. (2002) 23:303–8. PMID: 12195231

[256] Mizutani M Ihara T Kaziwara K. Effects of orally administered thiamine tetrahydrofurfuryl disulfide on foetal development of rabbits and monkeys. Jpn J Pharmacol. (1972) 22:115–24. doi: 10.1254/jjp.22.115, PMID: 4624711

[257] Oi Y Shishido C Wada K Odaka H Ikeda H Iwai K. Allylthiamindisulfide and related compounds enhance thermogenesis with increasing noradrenaline and adrenaline secretion in rats. J Nutr Sci Vitaminol (Tokyo). (1999) 45:643–53. doi: 10.3177/jnsv.45.643, PMID: 10683815

[258] Narat JK Loef JA. Effects of a vitamin B1 concentrate. Arch Intern Med. (1937) 60:449–53. doi: 10.1001/archinte.1937.00180030066006

[259] Romanenko AV. Effect of thiamine on various types of synaptic junctions. Neirofiziol Neurophysiol. (1986) 18:621–9. 3022166

